# The cytoplasmic domain of the AAA+ protease FtsH is tilted with respect to the membrane to facilitate substrate entry

**DOI:** 10.1074/jbc.RA120.014739

**Published:** 2020-11-23

**Authors:** Vanessa Carvalho, Irfan Prabudiansyah, Lubomir Kovacik, Mohamed Chami, Roland Kieffer, Ramon van der Valk, Nick de Lange, Andreas Engel, Marie-Eve Aubin-Tam

**Affiliations:** 1 Department of Bionanoscience, Kavli Institute of Nanoscience, Delft University of Technology, Delft, the Netherlands; 2 BioEM Lab, C-CINA, Center for Cellular Imaging and NanoAnalytics, Biozentrum, University of Basel, Basel, Switzerland

**Keywords:** ATP-dependent protease, membrane protein, protein structure, electron microscopy, conformational change, cryo-EM, cryo-electron microscopy, LMNG, lauryl maltose neopentyl glycol, SEC, size-exclusion chromatography, SEC-MALS, size-exclusion chromatography combined with static light scattering, TEM, transmission electron microscopy

## Abstract

AAA+ proteases are degradation machines that use ATP hydrolysis to unfold protein substrates and translocate them through a central pore toward a degradation chamber. FtsH, a bacterial membrane-anchored AAA+ protease, plays a vital role in membrane protein quality control. How substrates reach the FtsH central pore is an open key question that is not resolved by the available atomic structures of cytoplasmic and periplasmic domains. In this work, we used both negative stain TEM and cryo-EM to determine 3D maps of the full-length *Aquifex aeolicus* FtsH protease. Unexpectedly, we observed that detergent solubilization induces the formation of fully active FtsH dodecamers, which consist of two FtsH hexamers in a single detergent micelle. The striking tilted conformation of the cytosolic domain in the FtsH dodecamer visualized by negative stain TEM suggests a lateral substrate entrance between the membrane and cytosolic domain. Such a substrate path was then resolved in the cryo-EM structure of the FtsH hexamer. By mapping the available structural information and structure predictions for the transmembrane helices to the amino acid sequence we identified a linker of ∼20 residues between the second transmembrane helix and the cytosolic domain. This unique polypeptide appears to be highly flexible and turned out to be essential for proper functioning of FtsH as its deletion fully eliminated the proteolytic activity of FtsH.

Cells are complex systems that rely on numerous tightly regulated vital processes. For instance, protein quality control is crucial for maintaining the cell’s proteome. To avoid the lethal accumulation of misfolded or nonfunctional proteins, eukaryotes as well as prokaryotes use proteolysis (1). In this process, peptide bonds are cleaved by proteases and the resulting amino acids (aa) are recycled to build new and functional proteins. This cycle allows cells to maintain their homeostasis. It is then understandable that malfunctions in proteolysis lead to diverse forms of disease (2, 3).

AAA+ proteases belong to the family of ATPases associated with various cellular activities and are molecular machines capable of unfolding and degrading proteins (4). AAA+ proteases share several structural and functional characteristics. They assemble into a barrel-shaped chamber with a central pore formed by the ATP-binding domains. The pore entrance exhibits translocating loops with highly conserved residues, which bind to target substrates. ATP-driven conformational changes of the ATP-binding domain unfold the bound substrate and translocate it through the central pore into the proteolytic chamber for degradation. In general, bacterial AAA+ protease malfunctions can lead to a complete discoordination of the cell homeostasis.

From the five AAA+ proteases in *Escherichia coli*, FtsH is the only one that is anchored to the membrane and that is essential (5). FtsH plays a crucial role in membrane protein quality control (6) and in aminoglycoside antibiotic resistance, possibly by eliminating misfolded proteins disruptive to the membrane (7). FtsH also regulates the phospholipid to lipopolysaccharide ratio in the outer membrane by degrading LpxC, the key enzyme of lipopolysaccharide biosynthesis (8).

In mitochondria, the FtsH ortholog called i-AAA protease translocates polynucleotide phosphorylase into the intermembrane space (9), whereas the hydrophobicity of a specific transmembrane segment dictates its dislocation from the inner membrane by the mitochondrial *m*-AAA protease, another FtsH ortholog (10). In humans, mutations in the gene coding for paraplegin, a subunit of *m*-AAA, are related to the severe disease *spastic paraplegia* (11). Therefore, and because the FtsH mechanics and structure are less well understood than those of cytoplasmic AAA+ proteases, increasing our knowledge on the mechanisms of FtsH is of both medical and fundamental interest.

The FtsH protein comprises an N-terminal transmembrane helix, an ∼75-aa periplasmic domain, a second transmembrane helix (12), and the larger cytoplasmic AAA+ and protease domains (13). FtsH proteins assemble into hexamers, with 12 transmembrane helices inserting into the lipid bilayer. The ATPase domain has conserved arginine residues that compose the second region of homology, which is believed to be crucial for FtsH oligomerization. This domain also houses the highly conserved Walker A and Walker B domains, which bind and hydrolyze nucleotides (13, 14).

Structural studies have used truncated FtsH forms with only the soluble C-terminal (cytosolic) part (13, 14, 15, 16, 17, 18, 19, 20) or with only the periplasmic domain (12). The single full-length structure known concerns *m*-AAA, the yeast mitochondrial ortholog of bacterial FtsH, which has been resolved at 12 Å resolution by cryo-electron microscopy (cryo-EM) (21). Therefore, no information on the conformational rearrangement of full-length FtsH in relation to the membrane when bound to nucleotides or to a substrate is available. Crystal structures of the cytosolic domain of FtsH exhibit a six- (13), two- (14, 15), or threefold (18) symmetric conformation of the ATPase domain. These different conformations suggest that the ATPase domain could move polypeptides in steps as long as 45 Å into the central cavity during ATP hydrolysis cycles (13). In contrast, the C-terminal protease domain always shows a sixfold symmetry for all crystal structures, *i.e.*, the cytosolic domain of *Thermus thermophiles* FtsH (19), *Thermotoga maritima* FtsH (13, 15), and *Aquifex aeolicus* FtsH (14, 18). The proposed mechanism for substrate entry in *m*-AAA is based on substrate recognition by solvent-exposed lateral regions of the FtsH cytosolic domain. Accordingly, a 13-Å gap between the membrane and the cytosolic domain observed by cryo-electron microscopy would provide access to substrate, which implies that only (partly) unfolded proteins can reach the translocating loops and be moved through the pore for degradation (21).

Here we report the first full-length structure of *A. aeolicus* FtsH (AaFtsH), which we determined with negative stain electron microscopy to a resolution of 20 Å and with cryo-electron microscopy to resolutions of 6.6 Å with sixfold symmetry and 16 Å without the symmetry imposed. Unexpectedly, upon detergent solubilization we found not only AaFtsH hexamers but also fully stable and active AaFtsH dodecamers. The dodecamer structure from negatively stained specimen was solved to a resolution of 25 Å, showing two AaFtsH hexamers sharing a single lauryl maltose neopentyl glycol (LMNG) micelle. This arrangement induces a tilt of the periplasmic domain with respect to the cytosolic domain. In this conformation, the periplasmic domain of one hexamer touches the cytosolic domain of the other hexamer. Cryo-EM analysis revealed a variety of S-shaped or V-shaped dodecameric structures with differing N-terminal interactions at resolutions from 12.3 to 20.5 Å.

Since both AaFtsH hexamers and dodecamers have similar ATPase and proteolytic activities, we propose that the cytosolic domain tilts with respect to the membrane plane so that substrates can reach the translocating pore loops, as required for substrate unfolding and degradation. Such a large conformational change relates to the unique properties of the 20-aa linker between the end of the second transmembrane helix and the ATPase domain. Eliminating this linker leads to inactive AaFtsH hexamers that are not able to form dodecamers.

## Results

### Full-length AaFtsH purification

**Figure 1:**
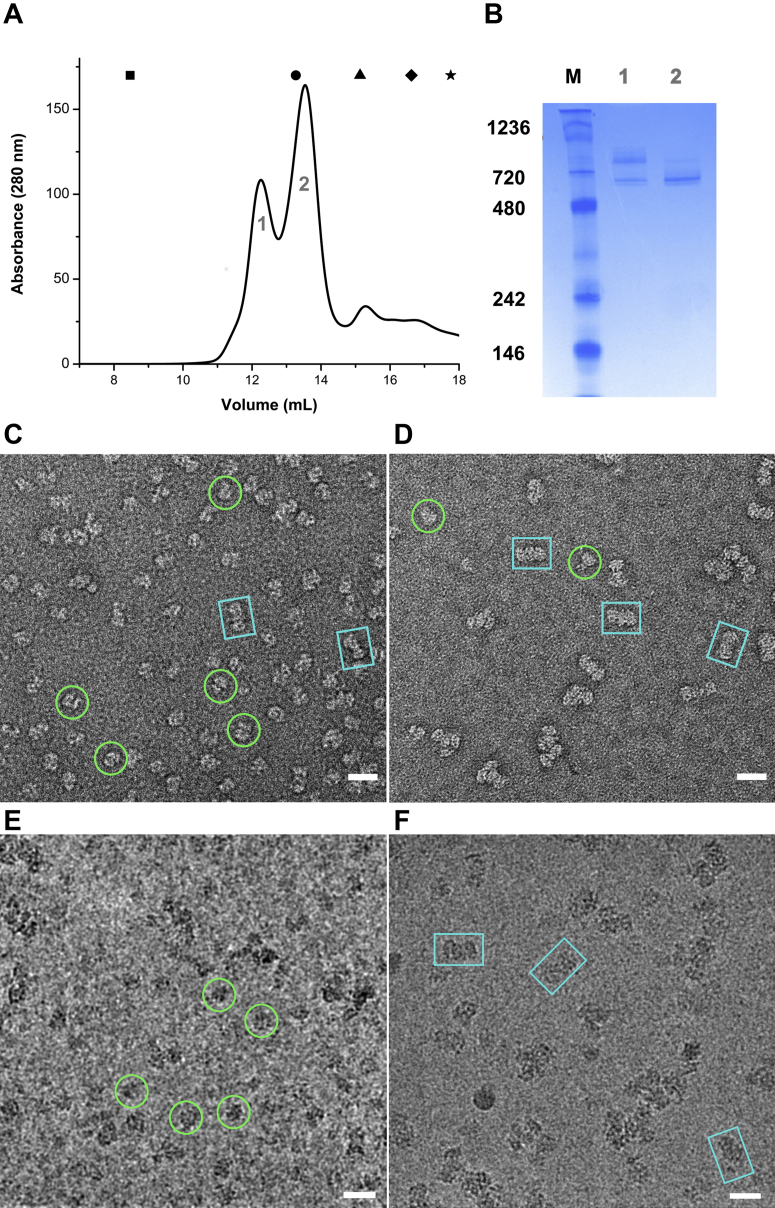
**FtsH oligomers elute in two peaks from sizing chromatography.***A*, representative size exclusion chromatography run. *Blue Dextran* (▪) used to calculate the void volume of the column (8.47 ml). The elution volumes of standard proteins used to estimate the molecular weight of the eluted fractions are shown on the top of the graph: (•) thyroglobulin (MW: 669 kDa; V_e_: 13.27 ml), (▲) ferritin (MW: 440 kDa; V_e_: 15.13 ml), (♦) aldolase (MW: 158 kDa; V_e_: 16.62 ml), and (★) ovalbumin (MW: 44 kDa; V_e_: 17.76 ml). *B*, native PAGE gel of peak 1 and 2 (lanes 1 and 2) and molecular marker (M). *C*–*D*, micrographs of the second (*C*) and first (*D*) SEC eluted fractions prepared by negative staining. Side views are preferentially obtained. *E*–*F*, sample cryo-EM micrographs of the hexameric (*E*) and dodecameric (*F*) specimens, with a few highlighted particles. The scale bars represent 20 nm. *Green circles* correspond to hexamers particles and *blue squares* to dodecamers.

AaFtsH with a C-terminal His-tag was overexpressed in *E. coli* cells and extracted from purified membranes with the use of a mild detergent, LMNG (22). Ni-NTA chromatography followed by size-exclusion chromatography (SEC) using Superose 6 10/300 GL yielded pure AaFtsH complexes (Fig. 1). Best results were obtained by incubating the AaFtsH-containing fractions, collected from Ni-NTA chromatography, overnight at 60 °C in the presence of 20 mM ATP, 10 mM MgCl_2_, and 25 μM ZnCl_2_ before the SEC purification. The SEC profile shows a first peak that is centered at 12.1 ± 0.2 ml (SD, N = 10) and a second peak at 13.4 ± 0.2 ml (SD, N = 10) (Fig. 1*A*). The peak positions were determined by simultaneously fitting two Gaussian functions. Native gel electrophoresis shows that the second peak has an approximate molecular weight of ∼700 kDa, whereas the first peak indicates a larger complex (Fig. 1*B*). The molecular weight of the eluted complexes was estimated using the partition coefficient (K_av_) values extracted from a calibration curve of the Superose 6 column and the positions of fitted Gaussian functions. The second peak is centered at a molecular weight of 730 kDa, which is larger than the size expected for AaFtsH hexamers (∼430 kDa), as expected owing to the weight contribution of the bound detergent micelle. On extrapolation of the calibration curve to smaller elution volumes, the first peak corresponds to a molecular weight of 940 kDa.

To determine more precisely the molecular weight of AaFtsH oligomers and LMNG micelles, size-exclusion chromatography combined with static light scattering (SEC-MALS) was used. SEC-MALS accounts for the amount of detergent bound to a membrane protein and allows for a determination of the molecular weight of a protein in a protein-detergent mixed micelle (23). SEC-MALS experiments of AaFtsH resulted in a molecular weight of 427 kDa for the protein in the second peak, which corresponds well to the molecular weight expected for an AaFtsH hexamer (430 kDa). The first peak has a molecular weight of 810 kDa, indicating a higher oligomeric state, close to the molecular weight expected for two hexamers. The molecular weight of LMNG micelle in the first and second peaks determined by SEC-MALS was 286 and 218 kDa, respectively (Fig. S1 and Table S1). These values are close to the molecular weight expected for LMNG micelles calculated with MALDI-TOF MS (24).

### The structure of AaFtsH hexamers

#### Negative stain EM

Transmission electron microscopy (TEM) was used to visualize samples from each SEC peak. Negative stain EM analysis of particles from the second peak, with molecular weight compatible with AaFtsH hexamers, exhibits structures with an average length of 154 ± 13 Å (SD, N = 280) (Fig. 1*C*).

**Figure 2:**
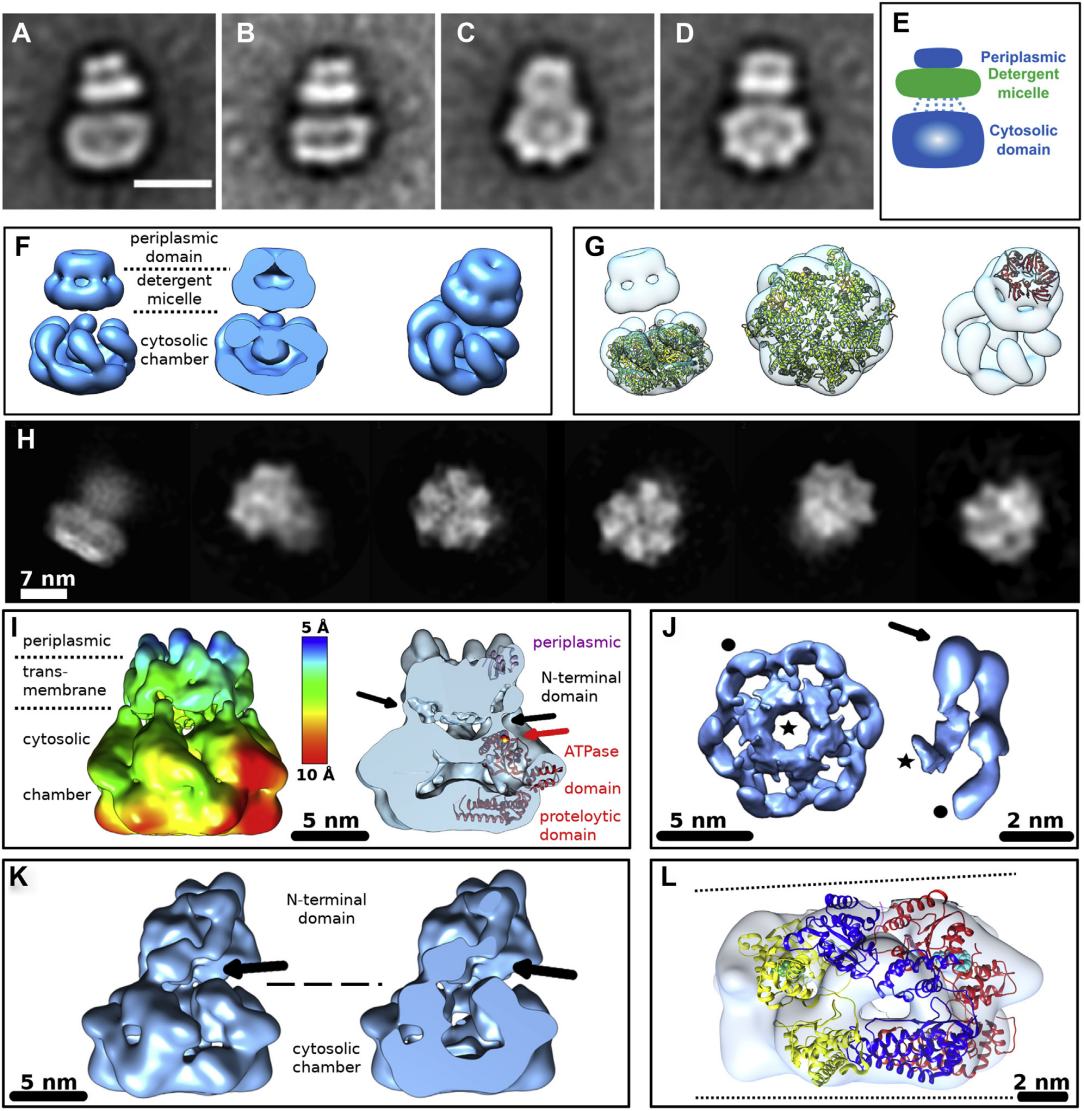
**Class averages and 3D maps of FtsH hexamers.***A*–*D*, class averages of the negatively stained specimen represent its side or tilted views. The scale bar represents 11 nm. *E*, schematic representation of the class averages comprising the periplasmic domain (*blue*), the transmembrane domain composed of 12 transmembrane helices kept in solution by the LMNG-micelle (*green*), and the cytosolic domain (*blue*). *Dashed blue lines* indicate six linking peptides present in the protein sequence. *F*, 3D maps calculated from images of negatively stained full-length AaFtsH hexamers. *G*, fitting of the crystal structure of the cytosolic domain of AaFtsH (PDB 4WW0) and the crystal structure of the periplasmic domain of *E. coli* FtsH (PDB 4V0B) to the hexamer map. *H*, left to right: cryo-EM class averages of the AaFtsH hexamer showing its side, top, and tilted views. *I*, left: structure of FtsH hexamer in C6 symmetry at the nominal resolution of 6.6 Å, colored according to local resolution analysis results. *I*, right: cross section through the structure in the vicinity of the C6 axis, showing two distinct connections of the N-terminal domain to the cytosolic domain (marked by *black arrows*). One fitted subunit of the FtsH X-ray cytosolic structure 4WW0 is shown in *red*, with a *red arrow* pointing at the position of its M141 residue. *Pink ribbons* indicate the position of the fitted 4V0B structure of the periplasmic domain. *J*, detailed views at the C6-symmetric N-terminal domain. Left: a top view along the C6 axis at a high threshold shows an inner and outer N-terminal ring; right: isolated N-terminal domain subunit forming a U-turn in the periplasmic domain (*arrow*). *Black star* and *dot* indicate connections to the cytosolic domain. *K*, left: a side view of the cryo-EM map of the AaFtsH hexamer resolved without a symmetry constraint at 16-Å resolution, displaying a tilted and disordered N-terminal domain. *K*, right: a cross section through the cryo-EM map. *Arrows* point at the opening into cytosol. *L*, positions of three of the YME1 protease subunits fitted into the asymmetric cytosolic chamber of FtsH. ADP-bound (6AZ0.E) is depicted in *yellow*, apo- (6AZ0.F) in *blue*, and ATP-bound (6AZ0.A) in *red*. ADP molecule is depicted as *green spheres*, ATP as *cyan*.

2D Class averages of the negative stain preparation were calculated from 15,000 images of AaFtsH hexamer particles using the image processing software packages Scipion1.1 (25) and EMAN2.12 (26). Figure 2, *A*–*D* displays representative side or tilted views of AaFtsH hexamers. Using 10 class averages from AaFtsH hexamers, we measured that the estimated length for the full hexamer is 167 ± 5 Å (SD, N = 10) and its width is 131 ± 7 Å (SD, N = 10). When compared with the dimensions of *A. aeolicus* FtsH cytosolic crystal structure, a similar width is reported (120 Å) (18). The cytosolic domain has a height of 83 ± 7 Å, and the periplasmic domain has a height of 31 ± 3 Å and a width of 63 ± 6 Å (Table S5). Finally, the detergent micelle, highlighted in green (Fig. 2*E*), has a thickness of 40 ± 4 Å, which is close to the lipid bilayer thickness, and width of 100 ± 18 Å.

From the 2D class averages, EMAN2.12 calculated starting models and refined one of them against all particles, imposing a sixfold symmetry. Figure 2*F* displays the 20-Å resolution 3D map of the AaFtsH hexamer, which accommodates the crystal structure of the AaFtsH cytosolic domain (PDB 4WW0) and the *E. coli* FtsH periplasmic domain (PDB 4V0B; Fig. 2*G*). Albeit the resolution achieved is only 20 Å, the orientation of the cytosolic domain with respect to the detergent micelle is clearly visible. Some of the hexamer classes show that the cytosolic domain is tilted in relation to the detergent micelle, creating a gap for substrate entry (Fig. 2, *A*–*D*).

#### Cryo-EM

Cryo-EM was then performed to visualize samples from the second SEC peak. 2D Class averages calculated from the initial dataset of 35,048 images of AaFtsH hexamer particles (Fig. 1*E*) using Relion 3.0.8 showed a large structural variability. In full-hexameric classes, the well-formed cytosolic domain displays high-resolution features (*e.g.*, C-terminal helices), whereas the N termini are fuzzy (Fig. 2*H*). Owing to a small number of particles remaining in the dataset after 3D classification (5649), 3D refinement was initially performed in C6 symmetry. The acquired 3D map revealed a protein structure similar to the negatively stained one, with clearly discernible cytosolic and N-terminal (periplasmic + transmembrane) domains (Fig. 2*I*, Table S5). The diameter of the cytosolic domain is 140 Å, length along the C6 axis is 134 Å, and the nominal FSC_0.143_ resolution determined by Relion is 6.6 Å. The cytosolic chamber could be fitted with six copies of the 4WW0 X-ray model comprising residues 141 to 608 in a similar way to the negatively stained 3D structure. The fitted 4WW0 subunits suggest the presence of a central orifice in the proteolytic domain, but the cryo-EM structure lacks it, likely owing to insufficient resolution and the introduction of the sixfold symmetry. On the other hand, the fitted ATPase domains form an ∼20-Å ring entrance into the proteolytic chamber, in agreement to structures of other proteases (20, 27, 28, 29). Adjacent to the first residue of the fitted crystal structures, M141, a narrow density extends from the cytosolic domain toward the inner part of the N-terminal domain and joins an inner ring of ∼40 Å outer diameter and ∼18 Å inner diameter, which is formed by all six N termini (Fig. 2*J*). From this ring, six symmetric densities extend further upward. Each of them then makes an outward U-turn of 2 × 90° and subsequently joins the cytosolic domain again on the outer surface. The crystallized 4V0B structure of the periplasmic domain could be fitted in the top of the U-turn density in the expected position between the two transmembrane helices (Figs. 2*I* and S2). However, the fit was unreliable (avg. CC = 0.0095 by rigid-body fitting in UCSF Chimera).

In order to investigate the dynamics of the structure, we performed a local resolution analysis in Relion, which indicated that the resolution varied from the highest 6.6 Å in the periplasmic domain to less than 10 Å in the cytosolic domain. This result, together with the presence of the closed channel in the cytosolic chamber (Fig. 2*I*), suggested that the symmetry constraint should be released.

Therefore, we also resolved the structure of the AaFtsH hexamer without imposing symmetry at 15.9 Å resolution, using only 2129 particles (Fig. 2*K*). It possesses a distorted cytosolic domain, which could still be fitted with six individual 4WW0 subunits. Its N-terminal domain is fully disordered, and only one subunit seems to partially follow the folding of the C6-symmetric structure. All six N termini bundle into an indistinguishable, tilted off-axis mass, which was already observed in the negatively stained class averages (Fig. 2, *A*–*D*). The N termini do not form any inner ring; instead, they give rise to a wide opening between the membrane and the cytosolic domain (Fig. 2*K*) for substrate entry.

Finally, we attempted to find out if the cytosolic domain of the asymmetric FtsH cryo-EM map possesses the staircase arrangement observed in other AAA ATPases (20, 27) with the help of the high-resolution structure of the YME1 protease (20). Its model (PDB ID: 6AZ0) describes an active state of YME1 during substrate processing, with four subunits bound to ATP, one to ADP, and one free of nucleotides, which gave rise to an asymmetric cytosolic domain (Figs. 1, 4, and 5 in (20)). We had incubated the FtsH with ATP; therefore, its individual subunits may be binding ATP, ADP, or no nucleotide. Since the amino acid sequences of the ATPase and proteolytic domains of *A. aeolicus* and the YME1 protease are highly similar, we attempted to fit the 6AZ0 model of the YME1 cytosolic domain into the cytosolic domain of FtsH cryo-EM map. As a result, the FtsH map and the YME1 model shared the asymmetric appearance induced by the nucleotide-dependent conformational changes (Fig. 2*L*, CC = 0.13 by rigid-body fitting in UCSF Chimera).

**Figure 3:**
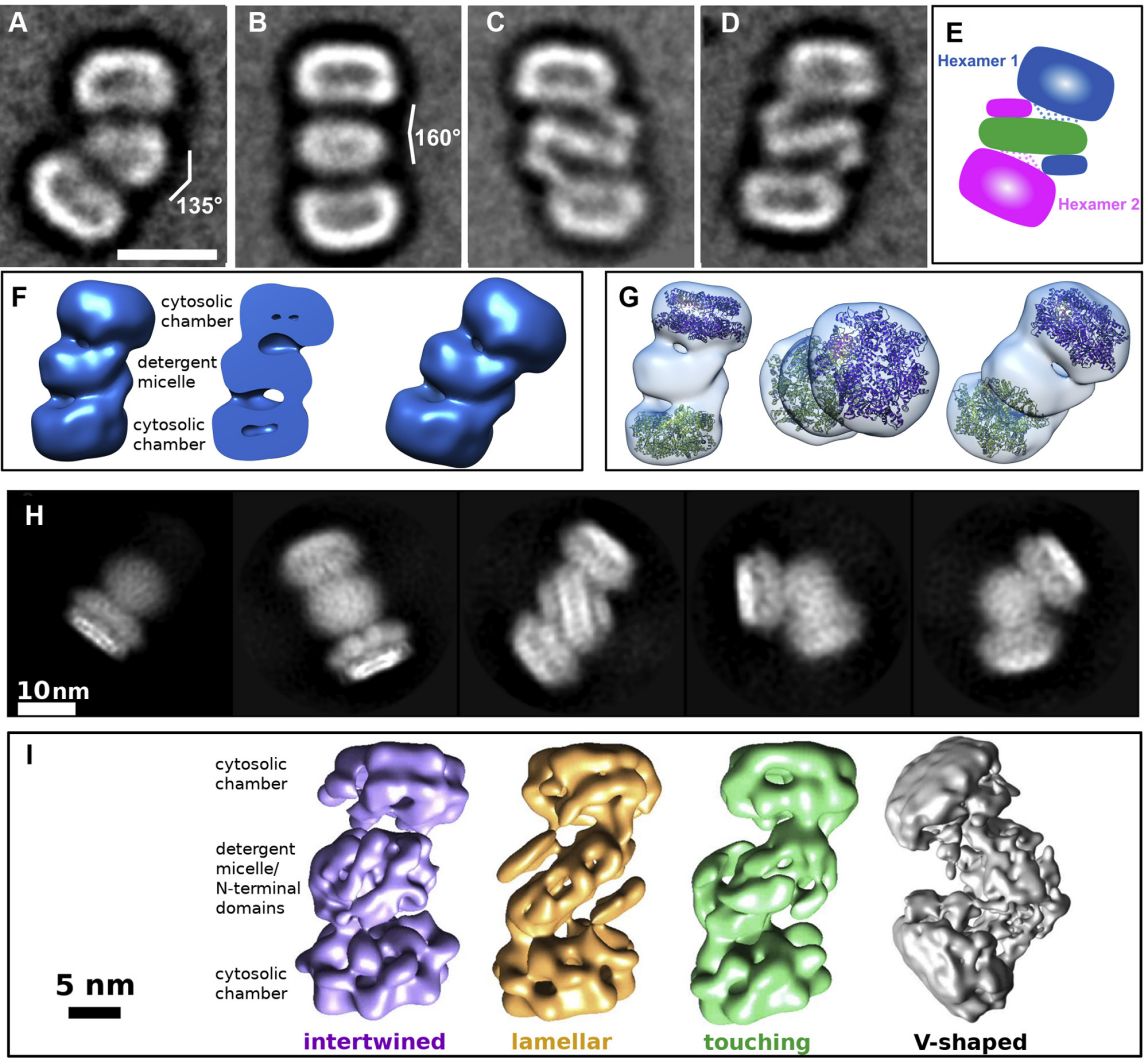
**Class averages and 3D maps of FtsH dodecamers.***A*–*D*, class averages of the negatively stained specimen representing its typical side views. The scale bar represents 11 nm. *E*, schematic representation of the class averages highlighting with dashed lines six linking peptides present in the protein sequence. *F*, 3D maps and a cross section of different dodecameric conformations calculated from images of the negatively stained specimen. *G*, fitting of two cytosolic domains (PDB 4WW0) to the dodecamer map. *H*, cryo-EM class averages of the AaFtsH dodecameric specimen. Left to right: a hexamer side view, followed by four typical side views of a dodecamer. *I*, left to right: S-shaped FtsH dodecamers with variably intertwined N-terminal domains at resolutions of 17 Å (intertwined type), 19.5 Å (lamellar type), and 20.5 Å (touching type), respectively, and a V-shaped dodecamer at 12.3 Å.

**Figure 4:**
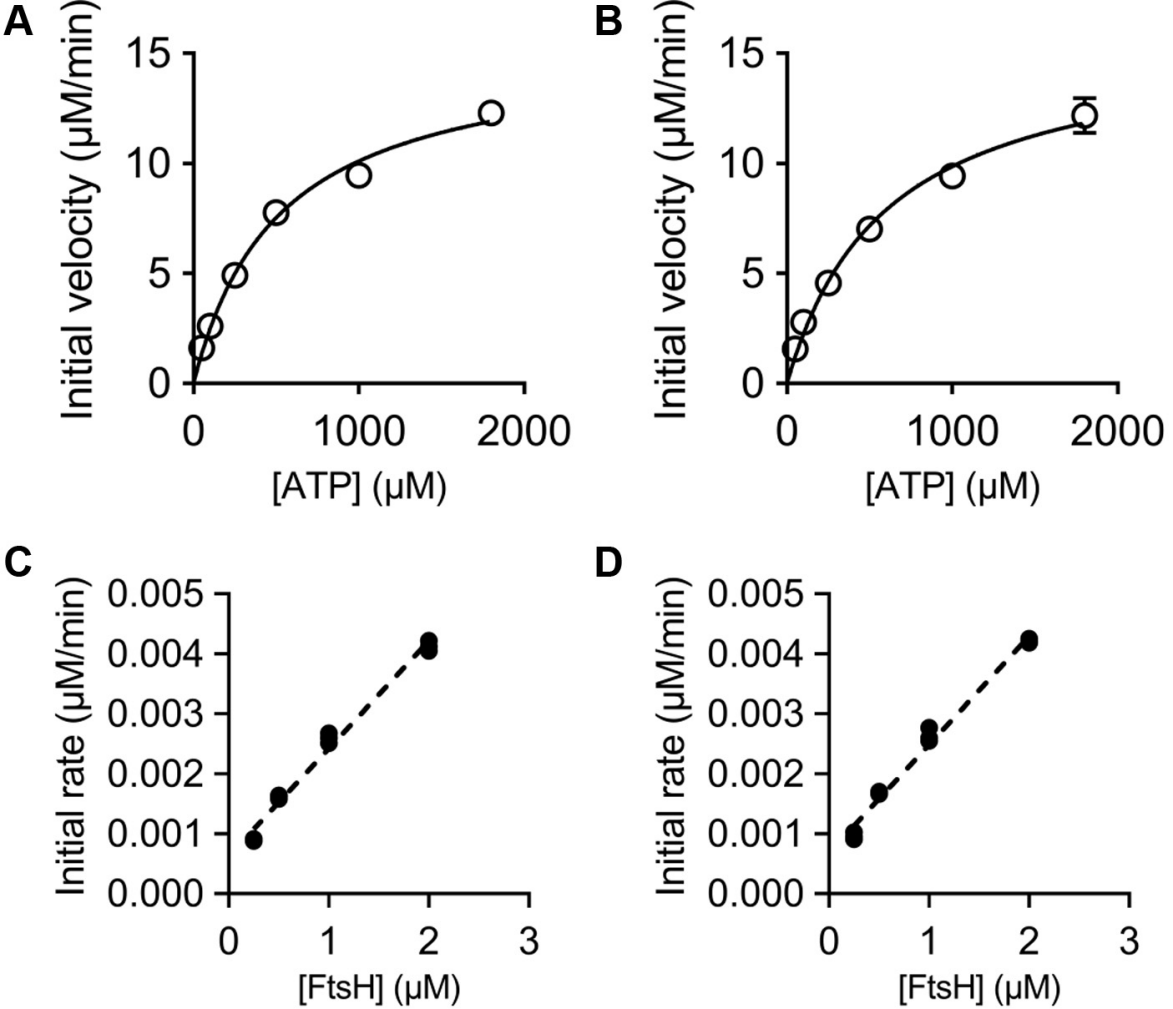
**ATPase and protease activity assays.***A*–*B*, steady-state velocities for the ATP hydrolysis by 0.25 μM AaFtsH hexamers (*A*) and dodecamers (*B*). The average and SD of three replicas for each reaction are plotted (SD bars are not visible for some points owing to their small sizes). *C*–*D*, initial velocities of proteolysis of 50 μM resorufin-labeled casein by AaFtsH hexamers (*C*) and dodecamers (*D*) as a function of AaFtsH concentration. All the reactions were measured three times and all points are plotted.

**Figure 5:**
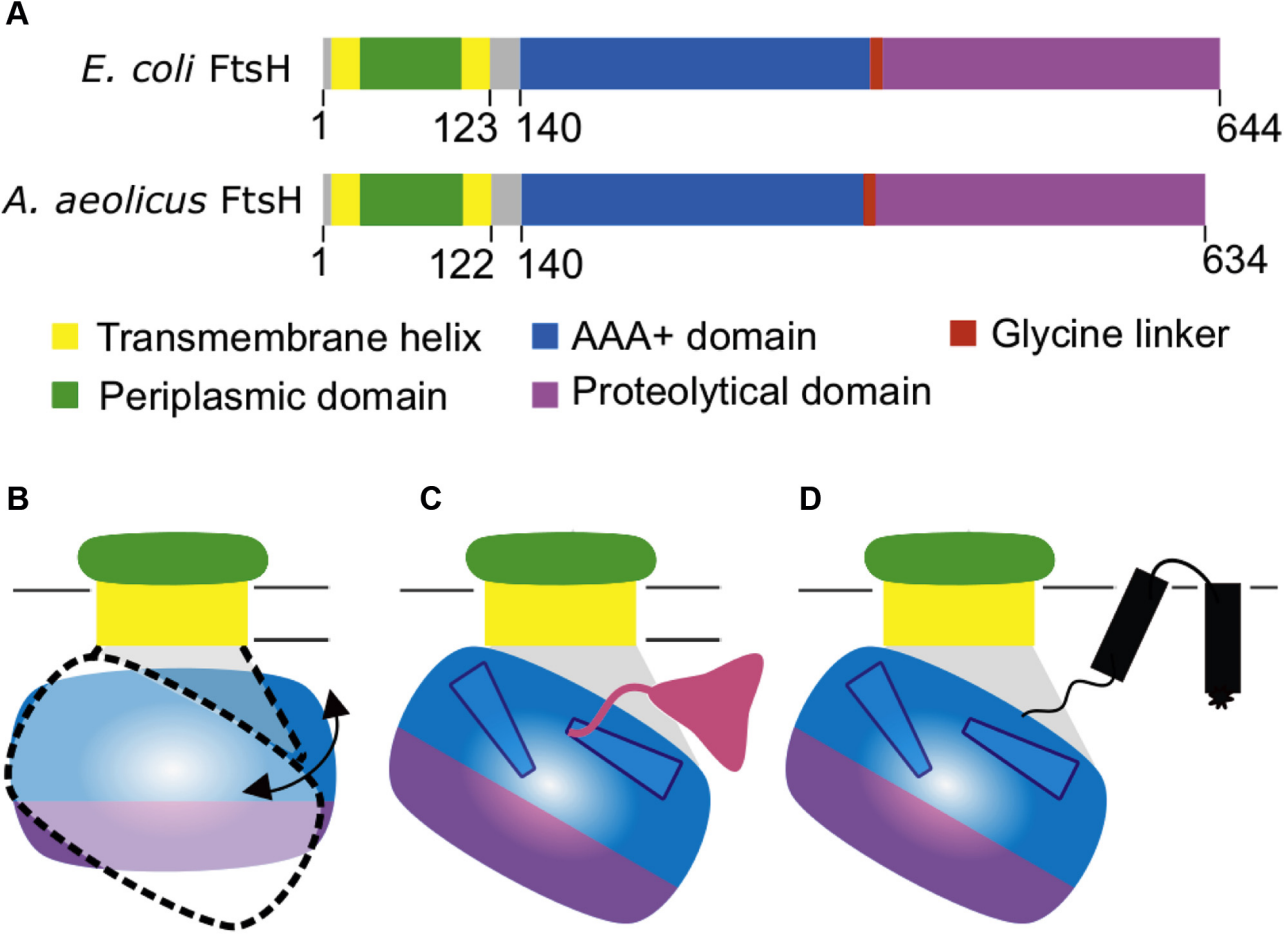
**Schematic representation of AaFtsH sequence and proposed model for substrate entry.***A*, bioinformatics tools and available structures of FtsH domains, from *E. coli* and *A. aeolicus*, show the presence of a loop-like peptide structure with ∼20 aa between membrane and AAA domains. The N-terminal periplasmic domain (*green*) is between two transmembrane helices (*yellow*). The second transmembrane helix is followed by a loop region (*gray*; see text), which is the link to the AAA+ domain (*blue*). Connected by the glycine linker (*red*), the C-terminal protease domain is shown in *purple*. *B*, a new model for substrate entry is proposed. The ∼20-aa flexible linker could allow the cytosolic domain of FtsH to tilt in relation to the membrane, creating a 30-Å wide gap that provides access of cytosolic (*pink*, *C*) and membrane protein substrates (*black*, *D*) to the central pore in a partially folded state.

### The structure of AaFtsH dodecamers

#### Negative stain EM

Negative stain EM was also performed on the first SEC peak revealing elongated particles with an average length of 231 ± 15 Å (SD, N = 280; Fig. 1*D*). Particles from the first peak appear to house two AaFtsH hexamers, hence corresponding to a dodecamer, although their average length is less than twice the average length of the hexamer particles.

2D Class averages were calculated using 15,000 images of AaFtsH dodecamer particles yielding class averages showing the conformational variability of AaFtsH (Fig. 3, *A*–*D*). To obtain a 3D map of the dodecamers, class averages that correspond to a straight (Fig. 3, *B*–*D*) rather than a V-shaped conformation (Fig. 3*A*) were selected to calculate an initial model that was subsequently refined against all projections of these classes without imposing any symmetry. Figure 3, *F*–*G* illustrate the resulting 3D map and indicate how the structure of the AaFtsH cytosolic domain (PDB 4WW0) fits. All class averages and 3D maps suggest that the AaFtsH dodecamer is kept in solution by a single LMNG micelle that embraces 2 × 12 transmembrane helices (green in Fig. 3*E*), whereas the periplasmic domains of each hexamer contact the cytosolic domains of the other hexamer (pink and blue in Fig. 3*E*).

Using 10 class averages from AaFtsH dodecamers, we measured that the detergent micelle has a thickness of 43 ± 2 Å and a width of 126 ± 7 Å. The estimated length of the dodecamer is 243 ± 8 Å, accommodating two tilted and distorted hexamers. The area that encompasses the micelle plus the two periplasmic domains is 90 ± 6 Å. All the dodecamer classes show that the cytosolic domain is tilted relative to the detergent micelle, as observed also in the hexamer classes (Fig. 2, *A*–*D*). A gap as large as 20 Å is observed between the central pore of the AaFtsH cytosolic domain and the detergent micelle, and a gap of ∼30 Å between the edge of that domain and the micelle (Fig. S3). This gap is large enough to accommodate the whole periplasmic domain of the second hexamer to which it is complexed.

#### Cryo-EM

The dodecamer structures resolved with cryo-EM confirm the structural variability observed in negatively stained 2D class averages (Fig. 3*H*). From the initial dataset of 101,726 particle images identified by Gautomatch picking (Fig. 1*F*), 41,818 remained after several Relion 2D classification runs. They were submitted to a 3D classification, which identified 25% of hexameric, 72% of dodecameric particles, and 3% misshaped particles. From the dodecameric particles, 36% contributed to two dodecameric structures connected at angles close to 90°, and 64% to one of three classes of S-shaped dodecamers (Fig. 3*I*), which seem to be composed of two opposing AaFtsH hexamers with tilted N-terminal domains.

Subsequent 3D classification of both V-shaped dodecameric classes (angled at 90°–110°, Fig. 3*I*) further accentuated the structural variability by assigning their particles into many low-resolution classes unsuitable for midresolution refinement. The final dataset of the S-shaped classes, comprising 16,749 particle images, could be split into three major classes with final counts of 4351, 2651, and 1722 particle images, which could be refined to resolutions of 17, 19.5, and 20.5 Å, respectively (Fig. 3*I*). These three classes strongly differ in the structure of the micellar domain, which can be described as “intertwined” (17 Å), “lamellar” (19.5 Å), and “touching” (20.5 Å). Owing to the low resolution, it is impossible to trace the course of individual N-terminal domains in the micellar part. On the other hand, one of the cytosolic domains is always better structured than its counterpart, which suggests further structural variability within the class averages. The length of the S-shaped AaFtsH dodecamers is about 290 Å.

### Full-length AaFtsH hexamers and dodecamers show similar ATPase and protease activity

ATP hydrolysis rates were assessed by measuring the inorganic phosphate (Pi) released. The initial velocity of the ATP hydrolysis reactions is extracted from the concentration of Pi released in the first 10-min interval (Fig. S4). From this, specific ATPase activities of 338 and 340 nmol/min/mg were calculated for AaFtsH hexamers and dodecamers, respectively. To further compare the activity of FtsH in the hexameric and dodecameric forms, Michaelis–Menten constants (*K*_*M*_ and *K*_cat_) were fitted using initial velocities calculated at various ATP concentrations (Fig. 4, *A*–*B*), with the use of FtsH monomer concentrations in the calculations (30, 31). The results of the fits show that the hexamer fraction has a *K*_*M*_ = 600 μM (95% confidence interval [CI]: 480–754 μM) and a *K*_cat_ = 63 min^−1^ (95% CI: 58–69 min^−1^). The dodecamer fraction has a *K*_*M*_ = 518 μM (95% CI: 432–625 μM) and a *K*_cat_ = 61 min^−1^ (95% CI: 57–66 min^−1^), identical to the values for the hexameric AaFtsH within error limits. ATPase activity was measured at 60 °C. The optimal growth temperature of *A. aeolicus* is 85 °C (32); however, LMNG-solubilized AaFtsH aggregates at 80 °C. To ensure that it is valid to perform the malachite green assay at 60 °C, a control experiment was performed to calculate the measured release of Pi in the absence of AaFtsH (Fig. S4).

Next, we assessed the proteolytic activity of the hexamer and the dodecamer forms of AaFtsH. As previously reported (14, 33), we used resorufin-labeled casein substrate (Roche) to test the proteolytic activity over a range of AaFtsH concentrations. The initial rates of proteolytic activity were calculated from the concentration of resorufin released in the first 30-min interval (Fig. S5). We assessed initial velocities for different AaFtsH monomer concentrations using the hexamer (Fig. 4*C*) and the dodecamer (Fig. 4*D*) fractions. The hexamers and the dodecamers reaction rates both increased linearly with AaFtsH concentrations at a slope of ∼1.8 nM of resorufin per minute per micromole of AaFtsH. This validates the idea that both fractions have the same proteolytical activity, suggesting that their active centers are not only well folded but also equally accessible for protein entrance.

To prove that the oligomeric states of AaFtsH are fixed over the course of the experiment, the SEC fractions from the hexamer peak and the dodecamer peak were collected and incubated with protease buffer for 30 min at 60 °C, mimicking the protease activity assay conditions. After the 30-min incubation, both fractions were reanalyzed by SEC. Our SEC result indicates that AaFtsH hexamers and dodecamers remain in their oligomeric states, at least over the course of 30 min (duration of protease activity assay), as shown in the Fig. S6.

### Bioinformatics tools identify a linker region of ∼20 aa

To gain a better understanding of the unexpected conformational flexibility of AaFtsH complexes, we compared the *E. coli* and *A. aeolicus* FtsH protein sequences and mapped the available crystal structures onto the sequence alignment (Fig. S2). Different transmembrane helix predictors identified both the N-terminal helix and the helix linking the periplasmic to the ATPase domain. A region of ∼20 aa remained between the second transmembrane helix and the cytosolic domain (Fig. 5*A*). Basic Local Alignment Search Tool (BLAST) (34) finds that this 20-aa sequence is unique to membrane-bound AAA+ proteases. When tested with different structure predictors, this region exhibits an extended loop conformation with a weak signal for a short helix at its end. Analysis of residue conservation by ConSurf (35) shows 13 conserved or highly conserved residues in this 20-aa region (Fig. S7).

### Effect of the deletion of the 20-aa flexible linker

To assess the importance of the flexible linker between the TM and the AAA domain, we constructed the AaFtsH-Δ20 mutant, in which a genomic sequence corresponding to a 20-aa linker (residues 123–142) was deleted. SEC-MALS experiments show that AaFtsH-Δ20 eluted in a single peak with a molecular weight of ∼416 kDa corresponding to a hexamer (theoretical mass of 417 kDa); this assembly was further confirmed by negative stain EM (Fig. S8 and Table S2). In contrast to the AaFtsH wildtype, the AaFtsH-Δ20 mutant does not produce dodecamers. This result confirms that deletion of the linker affects the conformational flexibility of AaFtsH. The proteolytic activity assays show that, although AaFtsH-Δ20 maintains its hexameric conformation, all proteolytical activity is lost, suggesting that the linker is crucial for the function of AaFtsH (Fig. S8).

## Discussion

In this work, we have purified full-length *A. aeolicus* FtsH and characterized its structure with negative stain and cryo-EM. The purification of full-length FtsH hexamers proved to be challenging, with size exclusion chromatograms reproducibly showing two species of AaFtsH in closely overlapping fractions. SEC-MALS and electron microscopy image analysis confirmed that the low-molecular-weight fraction corresponds to AaFtsH hexamers and the high-molecular-weight fraction to AaFtsH dodecamers (Figure 1, Figure 2, Figure 3 and S1). Importantly, both fractions show highly similar specific ATPase activities: 338 nmol/min/mg for AaFtsH hexamers and 340 nmol/min/mg for AaFtsH dodecamers. This compares well with values reported for the *E. coli* FtsH, 230 (31) and 193 nmol/min/mg (30). Also, the Michaelis–Menten constants are similar for the hexameric and dodecameric forms, with *K*_*M*_ (600 and 518 μM, respectively) and *K*_cat_ (63 and 61 min^−1^, respectively). The *K*_*M*_ values obtained for ATPase activity of hexamers and dodecamers are between 7× (31) and 20× (30) higher than reported for *E. coli* FtsH solubilized in a different detergent and under different conditions. Both fractions of LMNG-solubilized AaFtsH can degrade a casein substrate at comparable proteolytic rates (Fig. 4, *C*–*D*). Our results also document that AaFtsH hexamer and dodecamer both show high proteolytic activity, since the same amount of casein can be degraded by 10x less LMNG-AaFtsH when compared with AaFtsH solubilized in n-dodecyl-β-D-maltoside (14). Both AaFtsH hexamer and dodecamer are stable and remain in their oligomeric states during the activity assays period (Fig. S6). In summary, we demonstrate that the AaFtsH kept in solution by LMNG exists in a more stable form than previously reported and that they are equally active in hexameric and dodecameric forms.

Furthermore, we report here the first full-length bacterial FtsH 3D map, which we determined by negative stain and cryo-EM. As documented in Figure 2*G*, the negative stain EM map accommodates X-ray structures of cytosolic and periplasmic fragments. The density of the X-ray structure of *A. aeolicus* cytosolic domain (PDB 4WW0) rendered at 20-Å resolution fits well with the cytosolic domain of the 3D map presented here. There is a similar match of the periplasmic domain with the periplasmic crystal structure of *E. coli* FtsH (PDB 4V0B). The reported intermembrane domain of *m*-AAA is larger (21), as it comprises ∼25% more amino acids than the AaFtsH periplasmic domain.

The sixfold-symmetric cryo-EM map of the AaFtsH hexamer could accommodate both crystal structures in a similar way. The difference is that the individually fitted 4WW0 subunits form a convex surface of the cytosolic domain in the vicinity of the outlet rather than a plane. The local resolution analysis indicated that the periplasmic domain maintains the sixfold symmetry, whereas the cytosolic FtsH domain functions as an asymmetric machine, like related AAA+ proteases do (20, 27, 36, 37). The well-resolved periplasmic structure provided an insight into the organization of the N-terminal domain. In particular, the periplasmic domain seems to be U-turn shaped and connects to the cytosolic domain with two separated density streaks. Although this arrangement corresponds to the expected presence of two transmembrane helices (Fig. 5) (12), only one of the density connections can represent the amino acid sequence of the 20-aa linker between the end of the second transmembrane helix and the ATPase domain. The other connection likely represents the N terminus of the FtsH sequence in a close vicinity of the ATPase domain, rendered in the cryo-EM at insufficient resolution. The low resolution of the map also prevented us from distinguishing between the two connections.

Most surprisingly, however, the six inner N-terminal streaks connect into a ring, which could contribute to unfolding of substrates as demonstrated in the case of the Lon protease (38). The asymmetric hexamer structure of AaFtsH lacks such an inner ring. Instead, the N-terminal domains form a featureless tilted mass, which is impossible to trace. Despite this, the tilt gives rise to a wide opening into the cytosol that would facilitate substrate translocation. In addition, the cytosolic domain accommodates the asymmetric atomic model of the active YME1 cytosolic chamber well. Therefore, the asymmetric conformation is more likely the natural form of the AaFtsH hexamer, similarly to other ATPases (20, 27, 36, 37, 38, 39).

The unexpected AaFtsH dodecamers (Fig. 3, *A*–*D*, *H* and *I*) are also structurally highly variable, in particular considering the different arrangements of the N-terminal domains. Together with the well-resolved periplasmic domain (Fig. 2*I*), this indicates an amazing flexibility of the connector between periplasmic and cytosolic domains, providing FtsH with the capability to process a wide variety of substrates.

Although the 12-Å cryo-EM structure of *m*-AAA shows these thin links clearly (21), the subsequent model of the full-length *E. coli* FtsH predicts transmembrane helices projecting into the ATPase domain (12), which would not allow the conformational flexibility we observe. However, this model is compatible neither with the thin links shown by Lee *et al.* (21) nor with the significant stain-penetrated gap between the cytosolic AaFtsH domain and membrane domain visualized in Figure 2. In addition, our results suggest that the gap at the edge of the cytosolic domain of the native, hexameric AaFtsH may open up to ∼30 Å. Together, these observations have to be a consequence of the presence of a structure that enables such movement. Indeed, sequence analysis reveals a 20-aa linker present between the second transmembrane helix and the cytosolic domain sequence (Fig. 5*A*, gray), which exhibits a length of ∼13 Å in its quiescent state but can extend like a spring at least up to ∼30 Å, as the structure predictors show (Fig. S7). This linker’s possible extended loop conformation explains the structural rearrangements of the full-length FtsH in both hexameric (Fig. 2) and dodecameric (Fig. 3) forms. Moreover, the presence of an extended loop on the linker is compatible with the large movements of the ATPase domain predicted by X-ray crystallography (13). To further test the importance of this linker for FtsH function, we generated an AaFtsH mutant lacking the 20-aa linker (AaFtsH-Δ20). Our results show that the AaFtsH-Δ20 mutant does not produce dodecamers and is not functional (Fig. S8), which confirms that the linker is vital for FtsH conformational flexibility and its functionality. Together, these findings confirm our idea that the linker provides flexibility to allow for the insertion of substrates.

Based on these results we propose a new model for substrate entry. Conformational variations demonstrated by both AaFtsH hexamers and dodecamers (Figs. 2 and 3) indicate that FtsH is flexible enough for the large cytosolic domain to tilt significantly with respect to the membrane plane. At the same time, both forms maintain their activity levels. Therefore, it is likely that substrates enter the cytosolic domain sideways through the gap between the membrane and the cytosolic domain to be processed (Fig. 5, *B*–*D*). The space between the cytosolic domain and the tilted membrane compares with the height of the AaFtsH periplasmic domain. When FtsH adopts the tilted conformation, enabled by the flexible linker, both soluble and membrane-bound substrates would reach the translocation pore more efficiently (Fig. 5, *C*–*D*).

Although it is unlikely that FtsH forms head-to-tail dodecamers in the bacterial membrane, we propose that the large tilting movements observed here are likely achieved during proteolytic activity *in vivo*. A higher-resolution model of full-length FtsH would give us more details on how FtsH interacts with its substrates. This has, however, proven to be difficult, precisely owing to the high flexibility observed in the different particles present on EM grids.

## Experimental procedures

### AaFtsH expression and purification

Full-length *A. aeolicus* FtsH (AaFtsH) cloned into pET22a vector was kindly provided by Ulrich Baumann and the same expression conditions as previously reported were employed (14). Cells were harvested at 3500*g* for 25 min at 4 °C and disrupted using a French press. The resulting cell debris was purified at 20,000*g* for 15 min, and membranes were isolated at 125,000*g* for 3 h. Membranes were solubilized in 20 mM Tris-HCl pH 8.0, 150 mM NaCl, and 1% (w/v) LMNG (Anatrace) for 3 h at 4 °C and cleared at 125,000*g* for 1 h at 4 °C. The sample was first purified by affinity chromatography, using a HisTrap-5 ml column (GE Healthcare). FtsH fractions were eluted in 20 mM Tris-HCl pH 8.0, 500 mM NaCl, 0.01% (w/v) LMNG, and 200 mM imidazole. AaFtsH fractions were then incubated at 60 °C overnight with 20 mM ATP, 10 mM MgCl_2_, and 25 μM ZnCl_2_. The incubated sample was concentrated to 500 μl and loaded into a SEC Superose 6 Increase 10/300 GL column (GE Healthcare) pre-equilibrated with 10 mM Tris-HCl pH8.0, 150 mM NaCl, 0.01% (w/v) LMNG, and 5% glycerol. AaFtsH monomer concentration was measured with a Nanodrop. A representative SEC run is plotted in Figure 1*A*. The center position of the two largest peaks was determined by fitting Gaussian functions to 10 SEC profiles. A calibration curve of the Superose 6 Increase 10/300 GL was performed using the Gel Filtration High Molecular Weight Calibration Kit (GE Healthcare), following the GE Healthcare instructions. AaFtsH fractions were analyzed by Native PAGE gels using the MiniPROTEANTGX Precast Protein Gels (Bio-Rad).

To check the oligomeric state of AaFtsH over the course of the activity assays, the fractions corresponding to the first (dodecamers) and second (hexamers) fractions were incubated with the protease activity assay buffer (50 mM Tris-HCl pH 8.0, 80 mM NaCl, 12.5 μM ZnCl_2_, 5 mM MgCl_2_, 1 mM dithiothreitol, 0.01% LMNG, and 10 mM ATP) at 60 °C for 30 min. After that incubation period both fractions were reloaded into a SEC Superose 6 Increase 10/300 GL column (GE Healthcare) separately, the SEC column was pre-equilibrated with 10 mM Tris-HCl pH 8.0, 150 mM NaCl, 0.01% (w/v) LMNG, and 5% glycerol. The resulting SEC profile is in Fig. S6.

The AaFtsH-Δ20 mutant was generated by combining the PCR product formed by the AaFtsH_Primer 1 + 2 and AaFtsH_Primer 3 + 4 (Table S3), using the pET22a-AaFtsH plasmid as a template, with the aid of Gibson assembly (40). The construct was verified by DNA sequencing (Macrogen). AaFtsH-Δ20 was expressed and purified using the same procedure as that for the wildtype protein.

### SEC-MALS analysis

The molecular weight of AaFtsH oligomers and LMNG micelle were determined by SEC-MALS. SEC-MALS measurements were performed on a HPLC connected to a Wyatt DAWN-HELEOS instrument. The data were processed using the protein conjugate analysis program within the ASTRA software (Wyatt Technology). A dn/dc value of 0.14 ml/g was used for LMNG (41, 42). FtsH samples were run over a Superose 6 Increase 10/300 GL column in 20 mM Tris-HCl pH 8.0, 150 mM NaCl, and 0.01% LMNG at room temperature.

### Negative staining transmission electron microscopy analysis

Three microliters of AaFtsH hexamer or dodecamer fractions were loaded on a carbon-coated 400 square mesh copper grid (Aurion) previously glow discharged for 1 min. The liquid drop was absorbed with filter paper after 1 min and quickly washed with a drop of water that was again blotted with filter paper. This procedure was repeated three times to rinse all the detergent present in the samples. Finally, a 3-μl drop of 3% uranyl-acetate was added to the grid, incubated for 1 min and absorbed with a filter paper. TEM was performed using a Philips CM-200T and a JEOL 3200 FSC both equipped with a TemCam-F416 (TVIPS) and recorded at 50,000× magnification using the EM-MENU software with a sampling rate of 2.23 Å/pixel.

### Image processing of negatively stained single particles

Single particle imaging processing was performed using Scipion1.1 (25) and EMAN2.12 software packages (26). A set of 15,000 particles was manually picked using a semiautomatic mode using EMAN2.12. The particles were 2D averaged into 100 classes of which 50 well-structured 2D classes were selected as input for the calculation of the initial 3D model in EMAN2.12. This model was then refined against all particle images imposing the C6 symmetry. The resolution of the maps was calculated from the Fourier shell correlation (FSC) values as implemented in EMAN2.12. To measure the dimensions of the negatively stain particles, two approaches were taken. First, the height of hexamers and dodecamers was measured directly from 280 particles in the micrographs. Measurements referring to the different domains were done on 10 selected class averages. Using Chimera4.0.4 (43), different crystal structures of the *A. aeolicus* cytosolic domain (PDB 4WW0), the cryo-EM of the full-length *m*-AAA (EMDB 1712), and the crystal structure of the *E. coli* periplasmic domain (PDB 4V0B) were automatically fitted into our 3D map. To this end, the high-resolution structures were rendered to the resolution of the respective 3D maps. The fit of PDB 4WW0 to the FtsH cytosolic domain yielded a correlation of 0.6. Fitting the periplasmic crystal structure of *E. coli* (PDB 4VOB) to the periplasmic region achieved a correlation of 0.9. Similar correlation values were obtained when fitting two PDB 4WW0 to the 3D map of the FtsH dodecamer. The higher correlation values for the fits for the 3D maps of negatively stained samples compared with those for the 3D maps of vitrified samples relate the lower resolution of the former maps.

### Cryo-electron microscopy analysis

Three microliters of the purified AaFtsH samples at a concentration of 0.7 mg/ml were applied to a Quantifoil R1.2/1.3 (hexamers) or Quantifoil R2/2 300 mesh grid (dodecamers), glow discharged for 30 s prior to freezing. The grids were plunge frozen in liquid ethane with a FEI Vitrobot Mark IV (Thermo Fisher Scientific) under these conditions: temperature, 4 °C; humidity, 95%; blotting time, 2 s; and blotting force set to 1. Frozen grids were imaged in a Titan Krios TEM (300 kV, Thermo Fisher Scientific) equipped with a Gatan Quantum-LS energy filter (20-eV zero-loss filtering) and a Gatan K2 Summit detector. Images were acquired with SerialEM program (44) at total doses of 80 and 53 e^−^/Å^−2^ for hexamers and dodecamers, respectively. AaFtsH hexamers were imaged at a magnification of 47,259× (1.058 Å/pixel) and saved as 50-frame super-resolution stacks, dodecamers were imaged at a magnification of 78,247× (0.64 Å/pixel) and saved as counted stacks of 35 frames.

The acquired movies were processed during the imaging session with the Focus program (45), which included (i) gain reference application by the clip program from IMOD (46), (ii) motion correction and dose weighting by MotionCor2 (47), and (iii) contrast transfer function (CTF) estimation by CTFFIND4 (48). The super-resolution images of the hexamer sample were binned 2× by the resample_mp.exe program from Frealign (49) prior to motion correction. A total of 1371 aligned movies of the hexamer sample and 3993 of the dodecamer sample were used for further single-particle processing. Images displaying a resolution lower than 7 Å during CTF correction or average drifts higher than 1 Å per frame were excluded from the analysis.

### Image processing of cryo-EM datasets

Selection of particles from aligned micrographs was done in two steps. Initially, particles from 100 randomly selected images were semiautomatically selected with EMAN2 (50) and subjected to 2D classification in Relion 3.0 (51, 52). The resulting 2D class averages were then used as templates for automated particle picking with the Gautomatch program (https://www2.mrc-lmb.cam.ac.uk/research/locally-developed-software/zhang-software/#gauto). All calculations were performed by Relion 3.0.8. Table S4 contains all numbers related to image acquisition and image processing.

#### AaFtsH hexamers

A total of 35,048 AaFtsH particles were extracted from the aligned micrographs; 7635 particles were selected for subsequent 3D classification and refinement. In sixfold-symmetric processing, 5649 contributing to well-folded 3D classes were subjected to final 3D refinement, yielding a structure with 6.6-Å nominal resolution. Without an imposed symmetry, 3D classification revealed only one major class with 2129 particles, which could be refined to 15.9-Å resolution. A spherical blob with half-width of 140 Å was used as the initial 3D model.

#### AaFtsH dodecamers

A total of 101,726 particles were extracted from the dodecamer dataset; 41,818 were submitted to 3D processing with the same spherical blob serving as initial model. Five dodecameric structures, one hexameric structure, and one “rubbish” structure were identified in 3D classification with seven classes. Each of the dodecameric classes was further processed, only three “S-shaped” classes where the two AaFtsH hexamers seemed to be connected *via* their “tilted” N-terminal domains could be refined to resolutions of 17 Å (4351 particles), 19.5 Å (2651 particles), and 20.5 Å (1722 particles).

#### Fitting of X-ray and cryo-EM structures into cryo-EM maps

All rigid-body fitting was performed in UCSF Chimera. Although the resolved sixfold-symmetric hexamer structure could accommodate two copies of the 4WW0 trimeric FtsH crystal structure of *A. aeolicus*, better fit could be achieved when positioning the six 4WW0 subunits individually. The asymmetric 6AZ0 model of the YME1 protease was fitted in full length.

### ATPase activity

The ATPase activity was measured using the High Throughput Colorimetric ATPase Assays kit (Innova Biosciences). Free phosphate from the AaFtsH dodecamers and hexamers was eliminated by incubation with 100 μl of PiBind resin (Innova Bioscience) for 30 min. Also mix A (50 mM Tris-HCl pH 8.0; 150 mM NaCl; 10 mM MgCl_2_), was previously incubated with PiBind resin (Innova Bioscience). Reactions were started by mixing AaFtsH (0.25 μM final concentration) in mix A with 50, 100, 250, 500, 1000, or 1800 μM ATP (final concentrations). Reaction rates were measured every 2 min for a total of 10 min, in triplicates. ATP concentrations were chosen such that the maximum concentrations were more than 10× the previously reported *K*_*M*_ values (30, 31). Twenty-five microliters of P_i_ColorLock mix was added to stop the reaction, and after 5 min, 10 μl of stabilizer reagent was added. OD_650_ was measured after 30 min using a CLARIOstar (BMG-Labtech) microplate reader; every replica was measured three times and an average of these readings was calculated for each replica (Fig. S4). Released Pi concentrations were calculated from a calibration curve of standard Pi concentrations measured. *K*_*M*_ and *K*_cat_ were calculated for both hexamers and dodecamers. Michaelis–Menten constants were obtained, assuming the steady state of the reaction, with GraphPad Prism software with a 95% CI.

### Protease activity

AaFtsH protease activity was assessed using the resorufin-labeled casein kit (Roche); 0.25, 0.5, 1.0, or 2.0 μM (final concentrations) of AaFtsH hexamers or dodecamers were incubated with 50 μM of resorufin-labeled casein in (50 mM Tris-HCl pH 8.0, 80 mM NaCl, 12.5 μM ZnCl_2_, 5 mM MgCl_2_, 1 mM dithiothreitol, 0.01% LMNG, and 10 mM ATP) at 60 °C. Triplicates of these measurements were taken. Reaction rates were measured every 10 min for 30 min (Fig. S5). One hundred and sixty microliters of 5% trichloroacetic acid was added and incubated at 37 °C for 30 min. Proteins were precipitated at 16,100*g* for 30 min, and 120 μl from the supernatant was mixed with 80 μl of 500 mM Tris-HCl pH 8.8 and added to a 96-well transparent plate. The OD_574nm_ was immediately measured using an Infinite 200PRO (TECAN) plate reader.

### Sequence alignment and structure prediction

The AaFtsH and *E. coli* FtsH sequences were aligned with ESPript 3 (53). Part of the AaFtsH sequence (1–144 aa) was submitted to different transmembrane helices structure predictors: RHYTHM (54), CCTOP (55), TMHHM (56), HMMTOP (57), TOPCONS (58), and TMpred (Expasy) (59). Prediction of the transmembrane helices was performed to identify the beginning of the linker between membrane and AAA domains. To explore whether this 20-aa linker has a known structure, part of the sequence (110–148) was submitted to the structure predictors SCRATCH (60), PRE-FOLD 3 (61), and PREDICTPROTEIN (62). Sequence similarity search was performed with BLAST (blast.ncbi.nlm.nih.gov) (34). Residue conservation was assessed with the ConSurf server (35).

## Data availability

Cryo-EM structures of AaFtsH hexamers and dodecamers have been deposited under accession numbers EMD-11161, EMD-11167, EMD-11169, EMD-11171, EMD-11200, and EMD-11201. Other data from this study are available from the corresponding authors upon request.

## Conflict of interest

The authors declare that they have no conflicts of interest with the contents of this article.

## References

[1] Barrett A., Rawlings N., Woessner J. (2012). Handbook of Proteolytic Enzymes.

[2] López-Otín C., Bond J.S. (2008). Proteases: multifunctional enzymes in life and disease. J. Biol. Chem..

[3] Richard I. (2005). The genetic and molecular bases of monogenic disorders affecting proteolytic systems. J. Med. Genet..

[4] Olivares A.O., Baker T.A., Sauer R.T. (2016). Mechanistic insights into bacterial AAA+ proteases and protein- remodelling machines. Nat. Rev. Microbiol..

[5] Bittner L.M., Arends J., Narberhaus F. (2017). When, how and why? Regulated proteolysis by the essential FtsH protease in Escherichia coli. Biol. Chem..

[6] Hari S.B., Sauer R.T. (2016). The AAA+ FtsH protease degrades an ssrA-tagged model protein in the inner membrane of Escherichia coli. Biochemistry.

[7] Hinz A., Lee S., Jacoby K., Manoil C. (2011). Membrane proteases and aminoglycoside antibiotic resistance. J. Bacteriol..

[8] Schäkermann M., Langklotz S., Narberhaus F. (2013). FtsH-mediated coordination of lipopolysaccharide biosynthesis in Escherichia coli correlates with the growth rate and the alarmone (p) ppGpp. J. Bacteriol..

[9] Rainey R.N., Glavin J.D., Chen H., French S.W., Teitell M.A., Koehler C.M. (2006). A new function in translocation for the mitochondrial i-AAA protease Yme1: import of polynucleotide phosphorylase into the intermembrane space. Mol. Cell. Biol..

[10] Botelho S.C., Tatsuta T., von Heijne G., Kim H. (2013). Dislocation by the m-AAA protease increases the threshold hydrophobicity for retention of transmembrane helices in the inner membrane of yeast mitochondria. J. Biol. Chem..

[11] Nolden M., Ehses S., Koppen M., Bernacchia A., Rugarli E.I., Langer T. (2005). The m-AAA protease defective in hereditary spastic paraplegia controls ribosome assembly in mitochondria. Cell.

[12] Scharfenberg F., Serek-heuberger J., Coles M., Hartmann M.D., Habeck M., Martin J., Lupas A.N., Alva V. (2015). Structure and evolution of N-domains in AAA metalloproteases. J. Mol. Biol..

[13] Bieniossek C., Niederhauser B., Baumann U.M. (2009). The crystal structure of apo-FtsH reveals domain movements necessary for substrate unfolding and translocation. Proc. Natl. Acad. Sci. U. S. A..

[14] Vostrukhina M., Popov A., Brunstein E., Lanz M.A., Baumgartner R., Bieniossek C., Schacherl M., Baumann U. (2015). The structure of Aquifex aeolicus FtsH in the ADP-bound state reveals a C2-symmetric hexamer. Acta Crystallogr. D Biol. Crystallogr..

[15] Bieniossek C., Schalch T., Bumann M., Meister M., Meier R., Baumann U. (2006). The molecular architecture of the metalloprotease FtsH. Proc. Natl. Acad. Sci. U. S. A..

[16] Kim S.H., Kang G.B., Song H.E., Park S.J., Bea M.H., Eom S.H. (2008). Structural studies on *Helicobacter pylori* ATP-dependent protease, FtsH. J. Synchrotron Radiat..

[17] Niwa H., Tsuchiya D., Makyio H., Yoshida M., Morikawa K. (2002). Hexameric ring structure of the ATPase domain of the membrane-integrated metalloprotease FtsH from *Thermus thermophilus* HB8. Structure.

[18] Suno R., Niwa H., Tsuchiya D., Zhang X., Yoshida M., Morikawa K. (2006). Structure of the whole cytosolic region of ATP-dependent protease FtsH. Mol. Cell.

[19] Suno R., Shimoyama M., Abe A., Shimamura T., Shimodate N., Watanabe Y., Akiyama Y., Yoshida M. (2012). Conformational transition of the lid helix covering the protease active site is essential for the ATP-dependent protease activity of FtsH. FEBS Lett..

[20] Puchades C., Rampello A.J., Shin M., Giuliano C.J., Wiseman R.L., Glynn S.E., Lander G.C. (2017). Structure of the mitochondrial inner membrane AAA+ protease YME1 gives insight into substrate processing. Science.

[21] Lee S., Augustin S., Tatsuta T., Gerdes F., Langer T., Tsai F.T.F. (2011). Electron cryomicroscopy structure of a membrane-anchored mitochondrial AAA protease. J. Biol. Chem..

[22] Chae P.S., Rasmussen S.G.F., Rana R.R., Gotfryd K., Chandra R., Goren M.A., Kruse A.C., Nurva S., Loland C.J., Pierre Y., Drew D., Popot J., Picot D., Fox B.G., Guan L. (2010). Maltose–neopentyl glycol (MNG) amphiphiles for solubilization, stabilization and crystallization of membrane proteins. Nat. Methods.

[23] Slotboom D.J., Duurkens R.H., Olieman K., Erkens G.B. (2008). Static light scattering to characterize membrane proteins in detergent solution. Methods.

[24] Chaptal V., Delolme F., Kilburg A., Magnard S., Montigny C., Picard M., Prier C., Monticelli L., Bornert O., Agez M., Ravaud S., Orelle C., Wagner R., Jawhari A., Broutin I. (2017). Quantification of detergents complexed with membrane proteins. Sci. Rep..

[25] Rosa-trevín J. M. De, Quintana A., del Cano L., Zaldívar A., Foche I., Gutiérrez J., Gómez-blanco J., Burguet-castell J., Cuenca-alba J., Abrishami V., Vargas J., Otón J., Sharov G., Vilas J.L., Navas J. (2016). Scipion: a software framework toward integration, reproducibility and validation in 3D electron microscopy. J. Struct. Biol..

[26] Ludtke S.J. (2016). Single-particle refinement and variability analysis in EMAN2.1. Methods Enzymol..

[27] Botos I., Lountos G.T., Wu W., Cherry S., Ghirlando R., Kudzhaev A.M., Rotanova T.V., de Val N., Tropea J.E., Gustchina A., Wlodawer A. (2019). Cryo-EM structure of substrate-free E. coli Lon protease provides insights into the dynamics of Lon machinery. Curr. Res. Struct. Biol..

[28] Steele T.E., Glynn S.E. (2019). Mitochondrial AAA proteases: a stairway to degradation. Mitochondrion.

[29] Rotanova T.V., Andrianova A.G., Kudzhaev A.M., Li M., Botos I., Wlodawer A., Gustchina A. (2019). New insights into structural and functional relationships between LonA proteases and ClpB chaperones. FEBS Open Bio..

[30] Bruckner R.C., Gunyuzlu P.L., Stein R.L. (2003). Coupled kinetics of ATP and peptide hydrolysis by Escherichia coli FtsH protease. Biochemistry.

[31] Tomoyasu T., Gamer J., Bukau B., Kanemori M., Mori H., Rutman A.J., Oppenheim A.B., Yura T., Yamanaka K., Niki H., Hiraga S., Ogura T. (1995). *Escherichia coli* FtsH is a membrane-bound, ATP-dependent protease which degrades the heat-shock transcription factor sigma 32. EMBO J..

[32] Adams M.W.W., Kelly R.M. (1998). Finding and using hyperthermophilic enzymes. Trends Biotechnol..

[33] Akiyama Y. (2002). Proton-motive force stimulates the proteolytic activity of FtsH , a membrane-bound ATP-dependent protease in *Escherichia coli*. Proc. Natl. Acad. Sci. U. S. A..

[34] Altschul S.F., Madden T.L., Schäffer A.A., Zhang J., Zhang Z., Miller W., Lipman D.J. (1997). Gapped BLAST and PSI-BLAST: a new generation of protein database search programs. Nucleic Acids Res..

[35] Ashkenazy H., Abadi S., Martz E., Chay O., Mayrose I., Pupko T., Ben-tal N. (2016). ConSurf 2016: an improved methodology to estimate and visualize evolutionary conservation in macromolecules. Nucleic Acids Res..

[36] Śledź P., Unverdorben P., Beck F., Pfeifer G., Schweitzer A., Förster F., Baumeister W. (2013). Structure of the 26S proteasome with ATP-γS bound provides insights into the mechanism of nucleotide-dependent substrate translocation. Proc. Natl. Acad. Sci. U. S. A..

[37] Zhao M., Wu S., Zhou Q., Vivona S., Cipriano D.J., Cheng Y., Brunger A.T. (2015). Mechanistic insights into the recycling machine of the SNARE complex. Nature.

[38] Kereïche S., Kovacik L., Bednár J., Pevala V., Kunová N., Ondrovicová G., Bauer J., Ambro A., Bellová J., Kutejová E., Raška I. (2016). The N-terminal domain plays a crucial role in the structure of a full-length human mitochondrial Lon protease. Sci. Rep..

[39] Shin M., Puchades C., Asmita A., Puri N., Adjei E., Wiseman R.L., Karzai A.W., Lander G.C. (2020). Structural basis for distinct operational modes and protease activation in aaa+ protease lon. Sci. Adv..

[40] Gibson D.G., Young L., Chuang R.Y., Venter J.C., Hutchison C.A., Smith H.O. (2009). Enzymatic assembly of DNA molecules up to several hundred kilobases. Nat. Methods.

[41] Breyton C., Javed W., Vermot A., Arnaud C., Hajjar C., Dupuy J., Petit-hartlein I., Le Roy A., Martel A., Thépaut M., Orelle C., Jault J., Fieschi F., Porcar L., Ebel C. (2019). Assemblies of lauryl maltose neopentyl glycol (LMNG) and LMNG-solubilized membrane proteins. Biochim. Biophys. Acta Biomembr..

[42] Cooley R.B., O’Donnell J.P., Sondermann H. (2016). Coincidence detection and bi-directional transmembrane signaling control a bacterial second messenger receptor. Elife.

[43] Pettersen E.F., Goddard T.D., Huang C.C., Couch G.S., Greenblatt D.M., Meng E.C., Ferrin T.E. (2004). UCSF Chimera — a visualization system for exploratory research and analysis. J. Comput. Chem..

[44] Mastronarde D.N. (2005). Automated electron microscope tomography using robust prediction of specimen movements. J. Struct. Biol..

[45] Biyani N., Righetto R.D., McLeod R., Caujolle-Bert D., Castano-Diez D., Goldie K.N., Stahlberg H. (2017). Focus: the interface between data collection and data processing in cryo-EM. J. Struct. Biol..

[46] Kremer J.R., Mastronarde D.N., McIntosh J.R. (1996). Computer visualization of three-dimensional image data using IMOD. J. Struct. Biol..

[47] Zheng S.Q., Palovcak E., Armache J.P., Verba K.A., Cheng Y., Agard D.A. (2017). MotionCor2: anisotropic correction of beam-induced motion for improved cryo-electron microscopy. Nat. Methods.

[48] Rohou A., Grigorieff N. (2015). CTFFIND4: fast and accurate defocus estimation from electron micrographs. J. Struct. Biol..

[49] Grigorieff N. (2007). FREALIGN: high-resolution refinement of single particle structures. J. Struct. Biol..

[50] Tang G., Peng L., Baldwin P.R., Mann D.S., Jiang W., Rees I., Ludtke S.J. (2007). EMAN2: an extensible image processing suite for electron microscopy. J. Struct. Biol..

[51] Scheres S.H.W. (2012). RELION: implementation of a Bayesian approach to cryo-EM structure determination. J. Struct. Biol..

[52] Zivanov J., Nakane T., Forsberg B.O., Kimanius D., Hagen W.J.H., Lindahl E., Scheres S.H.W. (2018). New tools for automated high-resolution cryo-EM structure determination in RELION-3. Elife.

[53] Grosjean H., De Crécy-lagard V., Marck C. (2010). Deciphering synonymous codons in the three domains of life: Co-evolution with specific tRNA modification enzymes. FEBS Lett..

[54] Rose A., Lorenzen S., Goede A., Gruening B., Hildebrand P.W. (2009). RHYTHM — a server to predict the orientation of transmembrane helices in channels and membrane-coils. Nucleic Acids Res..

[55] Dobson L., Reményi I., Tusnády G.E. (2015). CCTOP: a consensus constrained TOPology prediction web server. Nucleic Acids Res..

[56] Krogh A., Larsson È., von Heijne G., Sonnhammer E.L.L. (2001). Predicting transmembrane protein topology with a Hidden Markov model: application to complete genomes. J. Mol. Biol..

[57] Tusnády G., Simon I. (2001). The HMMTOP transmembrane topology prediction server. Bioinformatics.

[58] Tsirigos K.D., Peters C., Shu N., Käll L., Elofsson A. (2015). The TOPCONS web server for consensus prediction of membrane protein topology and signal peptides. Nucleic Acids Res..

[59] Hofmann K., Stoffel W. (1993). TMbase: a database of membrane spanning protein segments. Biol. Chem. Hoppe-Seyler.

[60] Cheng J., Randall A.Z., Sweredoski M.J., Baldi P. (2005). SCRATCH: a protein structure and structural feature prediction server. Nucleic Acids Res..

[61] Lamiable A., Thévenet P., Rey J., Vavrusa M., Derreumaux P., Tufféry P. (2016). PEP-FOLD3: faster denovo structure prediction for linear peptides in solution and in complex. Nucleic Acids Res..

[62] Rost B., Yachdav G., Liu J. (2004). The PredictProtein server. Nucleic Acids Res..

