# Supplementary material for: The cytoplasmic domain of the AAA+ protease FtsH is tilted with respect to the membrane to facilitate substrate entry
Source: J Biol Chem. 2020 Nov 23;296:100029. doi: 10.1074/jbc.RA120.014739 (PMC7949044; doi:10.1074/jbc.RA120.014739)
Supplement: Supplementary Figures and Tables [file mmc1.pdf]

SUPPORTING INFORMATION

The cytoplasmic domain of the AAA+ protease FtsH is tilted with respect to the membrane to facilitate substrate entry

Vanessa Carvalho[1#], Irfan Prabudiansyah[1#], Lubomir Kovacik[2#], Mohamed Chami[2], Roland Kieffer[1], Ramon van der Valk[1], Nick de Lange[1], Andreas Engel[2]*, Marie-Eve Aubin-Tam[1]*

[1]Department of Bionanoscience, Kavli Institute of Nanoscience, Delft University of Technology. Van der Maasweg 9, Delft 2629 HZ, the Netherlands

[2]BioEM lab, C-CINA, Center for Cellular Imaging and NanoAnalytics, Biozentrum, University of Basel, Mattenstrasse 26, 4058 Basel, Switzerland.

Running title: *Conformational flexibility of the full-length FtsH*

[#] These authors contributed equally to this work.

*Corresponding authors: (Andreas Engel) andreas.engel@unibas.ch; (Marie-Eve Aubin-Tam) m.e.aubin-tam@tudelft.nl

CONTENTS

Figure S1. SEC-MALS analysis of AaFtsH.

Figure S2. Sequence alignment of the *E. coli* and *Aquifex aeolicus* FtsH full-length sequence.

Figure S3. Distance measurements between the cytosolic domain and the detergent micelle.

Figure S4. Inorganic phosphate release measurements.

Figure S5. Protease activity assay measurements.

Figure S6. SEC profiles of fractions 1 and 2 after purification and incubation in the protease activity assay buffer.

Figure S7. ~20 aa linker structure prediction

Figure S8. AaFtsH-Δ20 mutant characterization.

Table S1. Data derived from SEC-MALS of AaFtsH

Table S2. Data derived from SEC-MALS of AaFtsH-Δ20 mutant

Table S3. Primers used in this study

Table S4. FtsH image acquisition and image processing

Table S5. Dimensions of FtsH subunit

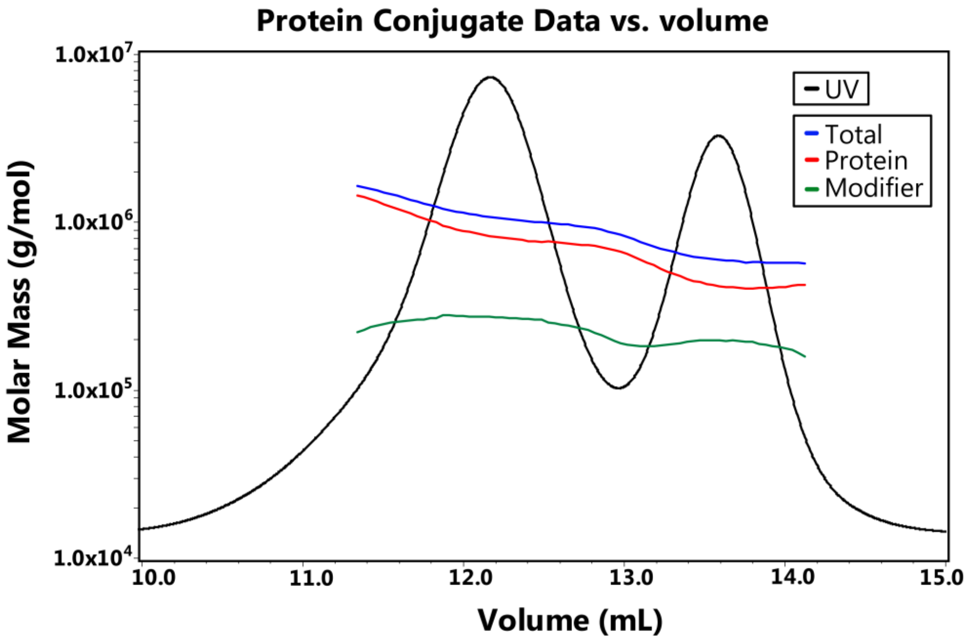

Figure S1. SEC-MALS analysis of AaFtsH. UV absorption chromatograms showing the distribution of the molar mass (g/mol) vs. retention volume (mL) for protein-detergent micelle. The calculated molar masses of protein, detergent micelle, and total of the protein-detergent micelle are shown in the supplementary Table S1.

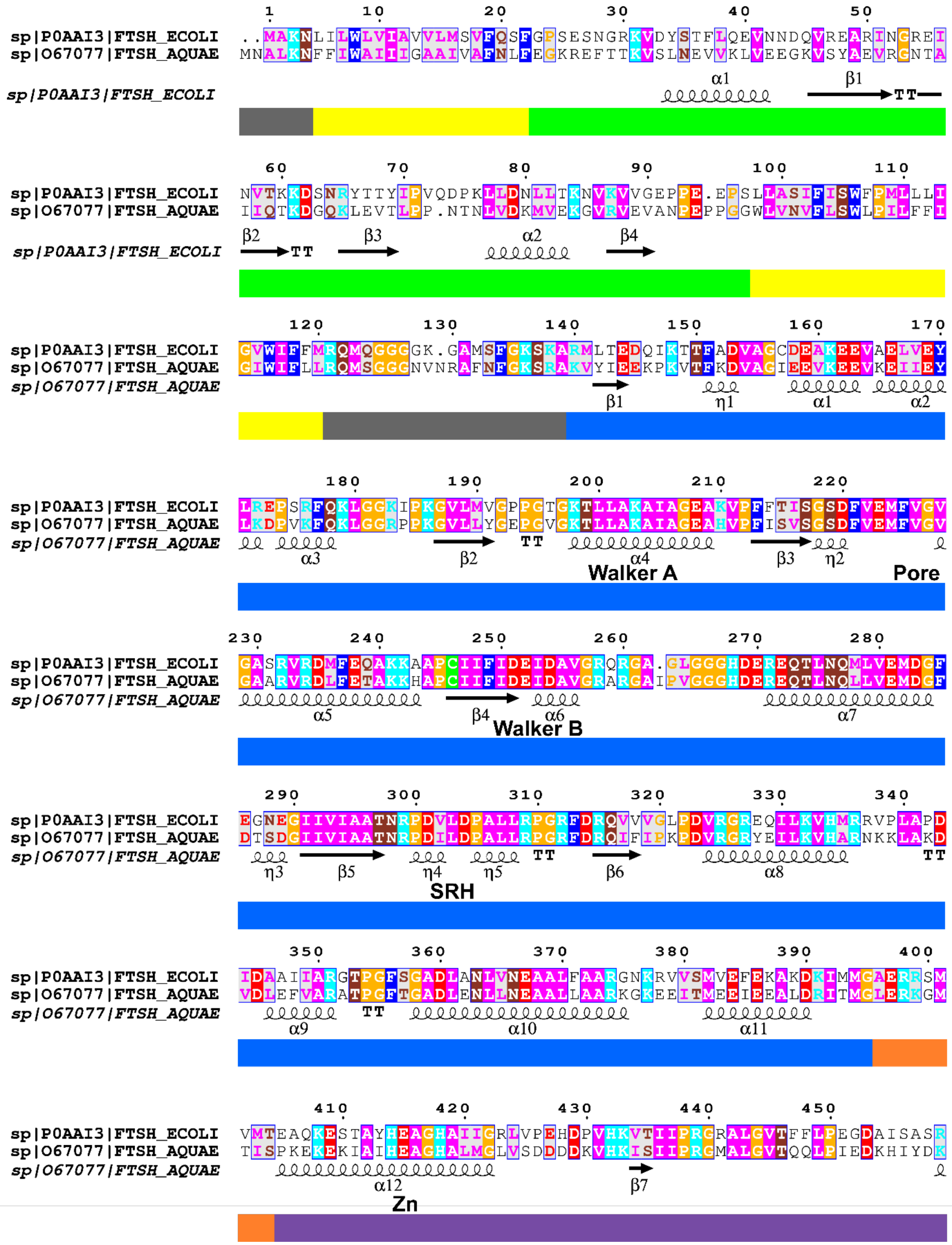
1 10 20 30 40 50
sp|P0AAI3|FTSH_ECOLI ..MAKNLILWLVIAVVLMSVFQSFGPSESNGRKVDYSTFLQEVNNDQVREARINGREI
sp|O67077|FTSH_AQUAE MNALKNFFIWAIIIGAAIVAFNLFEGKREFTTKVSLNEVVKLVEEGKVSYAEVRGNTA
sp|P0AAI3|FTSH_ECOLI α1 β1 TT
60 70 80 90 100 110
sp|P0AAI3|FTSH_ECOLI NVTKKDSNRYTTYIPVQDPKLLDNLLTKNVKVVGEPPE.EPSLLASIFISWFPMLLLI
sp|O67077|FTSH_AQUAE IIQTKDGQKLEVTLPP.NTNLVDKMVEKGVRVEVANPEPPGGWLVNVFLSWLPILFFI
sp|P0AAI3|FTSH_ECOLI β2 TT β3 α2 β4
120 130 140 150 160 170
sp|P0AAI3|FTSH_ECOLI GVWIFFMRQMQGGGGK.GAMSFGKSKARMLTEDQIKTTFADVAGCDEAKEEVAELVEY
sp|O67077|FTSH_AQUAE GIWIFLLRQMSGGGNVNRAFNFGKSRAKVYIEEKPKVTFKDVAGIEEVKEEVKEIIEY
sp|O67077|FTSH_AQUAE β1 η1 α1 α2
180 190 200 210 220
sp|P0AAI3|FTSH_ECOLI LREPSRFQKLGGKIPKGVLMVGPPGTGKTLLAKAIAGEAKVPFFTISGSDFVEMFVGV
sp|O67077|FTSH_AQUAE LKDPVKFQKLGGRPPKGVLLYGEPGVGKTLLAKAIAGEAHVPFISVSGSDFVEMFVGV
sp|O67077|FTSH_AQUAE α3 β2 TT α4 β3 η2
Walker A
Pore
230 240 250 260 270 280
sp|P0AAI3|FTSH_ECOLI GASRVRDMFEQAKKAAPCIIFIDEIDAVGRQRGA.GLGGGHDEREQTLNQMLVEMDGF
sp|O67077|FTSH_AQUAE GAARVRDLFETAKKHAPCIIFIDEIDAVGRARGAIPVGGGHDEREQTLNQLLVEMDGF
sp|O67077|FTSH_AQUAE α5 β4 α6 α7
Walker B
290 300 310 320 330 340
sp|P0AAI3|FTSH_ECOLI EGNEGIIVIAATNRPDVLDPALLRPGRFDRQVVVGLPDVRGREQILKVHMRRVPLAPD
sp|O67077|FTSH_AQUAE DTSDGIIVIAATNRPDILDPALLRPGRFDRQIFIPKPDVRGRYEILKVHARNKKLAKD
sp|O67077|FTSH_AQUAE η3 β5 η4 η5 TT β6 α8 TT
SRH
350 360 370 380 390 400
sp|P0AAI3|FTSH_ECOLI IDAAIIARGTPGFSGADLANLVNEAALFAARGNKRVVSMVEFEKAKDKIMMGAERRSM
sp|O67077|FTSH_AQUAE VDLEFVARATPGFTGADLENLLNEAALLAARKGKEEITMEEIEEALDRITMGLERKGM
sp|O67077|FTSH_AQUAE α9 TT α10 α11
410 420 430 440 450
sp|P0AAI3|FTSH_ECOLI VMTEAQKESTAYHEAGHAIIGRLVPEHDPVHKVTIIPRGRALGVTFFLPEGDAISASR
sp|O67077|FTSH_AQUAE TISPKEKEKIAIHEAGHALMGLVSDDDDKVHKISIIPRGMALGVTQQLPIEDKHIYDK
sp|O67077|FTSH_AQUAE α12 β7
Zn

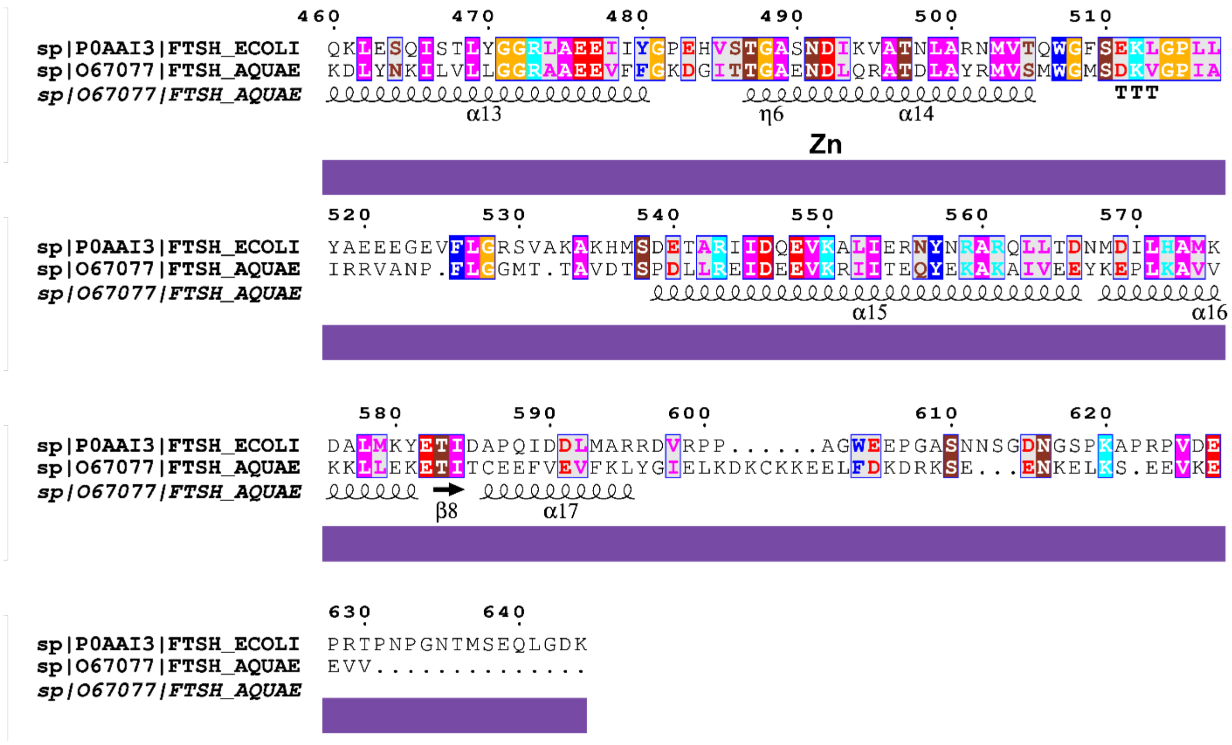

Figure S2. Sequence alignment of the *E. coli* and *Aquifex aeolicus* FtsH full-length sequence. Sequences in crystal structures of Aquifex aeolicus cytoplasm domain (PDB 4WW0) are underlined with coloured bars (AAA domain: blue; glycine linker: red; proteolytic domain: purple), and in the periplasmic domain (PDB 4V0B) with a green bar. Secondary structures are indicated by symbols below the sequence. Yellow bars refer to the two transmembrane helices and the grey bars correspond to the loop-like regions. Transmembrane helix modelling programs (RHYTHM, CCTOP, TMHHM, HMMTOP, TOPCONS and TMpred) predict the N-terminal helix (TM1) to contain 18 residues (FFIWAIIIGAAIVAFNLF) and the second helix (TM2) to contain 23 residues (WLVNVFLSWLPILFFIGIWIFLL). The alignment was performed using ESPript 3.

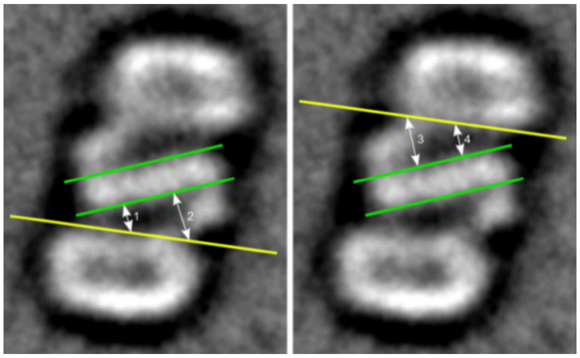

Figure S3. Distance measurements between the cytosolic domain and the detergent micelle. The distance between the central pore of AaFtsH cytosolic domain (delimited by the yellow line) and the detergent micelle (green lines) is approximately 20 Å (distances 1 and 4 are 18 Å and 20 Å, respectively), while ~30 Å (distances 2 and 3 are 32 Å and 33 Å, respectively) is measured between the edge of that domain and the micelle. This gap is large enough to accommodate partially folded proteins comparable in size to the periplasmic domain.

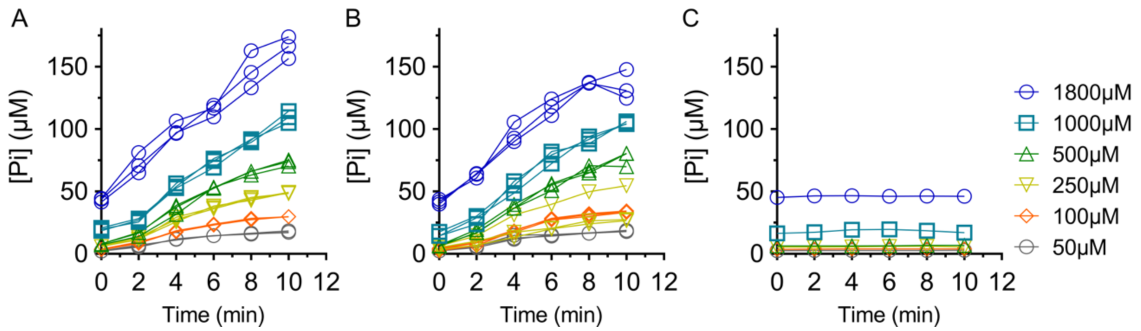

Figure S4. Inorganic phosphate release measurements. Free phosphate was measured using the Malachite Green assay kit for 0.25µM of AaFtsH hexamer (*A*) and dodecamer (*B*) fractions incubated with different ATP concentrations for 10 min. *C* – The free phosphate measurements in absence of AaFtsH.

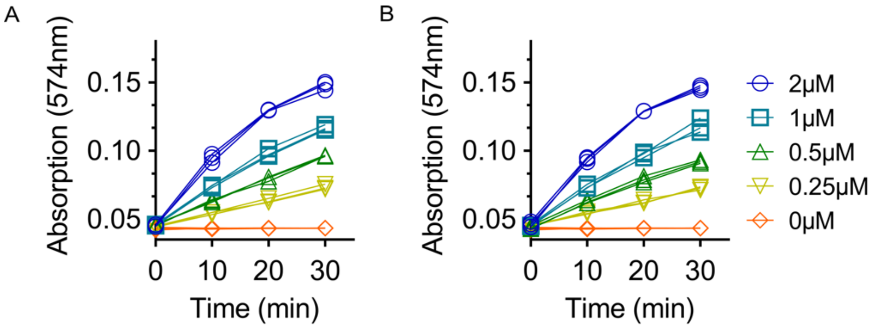

Figure S5. Protease activity assay measurements. Resorufin release was measured over 30 minutes for 50 µM of Resorufin labelled casein incubated with hexamer (*A*) and dodecamer fractions (*B*) at different concentrations.

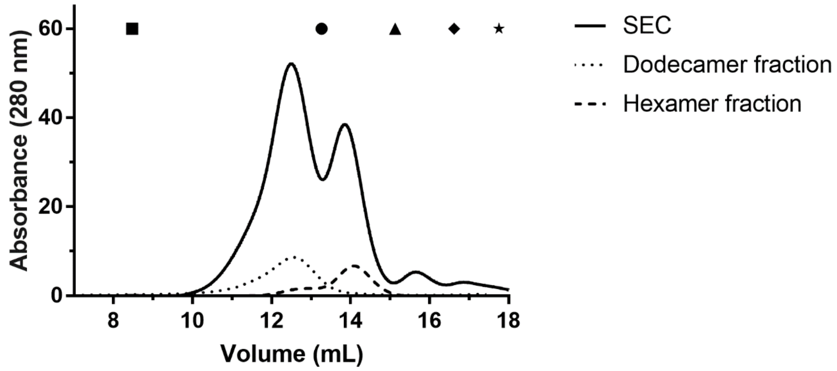

Figure S6. SEC profiles of fractions 1 and 2 after purification and incubation in the protease activity assay buffer. The original SEC profile for purification is shown as a solid line. The dotted lined profile corresponds to the re-running of the first fraction of the SEC, after incubation at 60 °C for 30 min in the protease activity assay buffer. The dashed lined profile corresponds to the re-running of the second fraction of the SEC, after incubation in the same conditions. Blue Dextran (■) used to calculate the void volume of the column (8.47mL). The elution volumes of standard proteins used to estimate the molecular weight of the eluted fractions are shown on the top of the graph: (●) Tyroglobulin (MW: 669 kDa; Ve: 13.27 ml), (▲) Ferritin (MW: 440 kDa; Ve: 15.13 ml), (♦) Aldolase (MW: 158 kDa; Ve: 16.62 ml) and (★) Ovalbumin (MW: 44kDa; Ve: 17.76 ml).

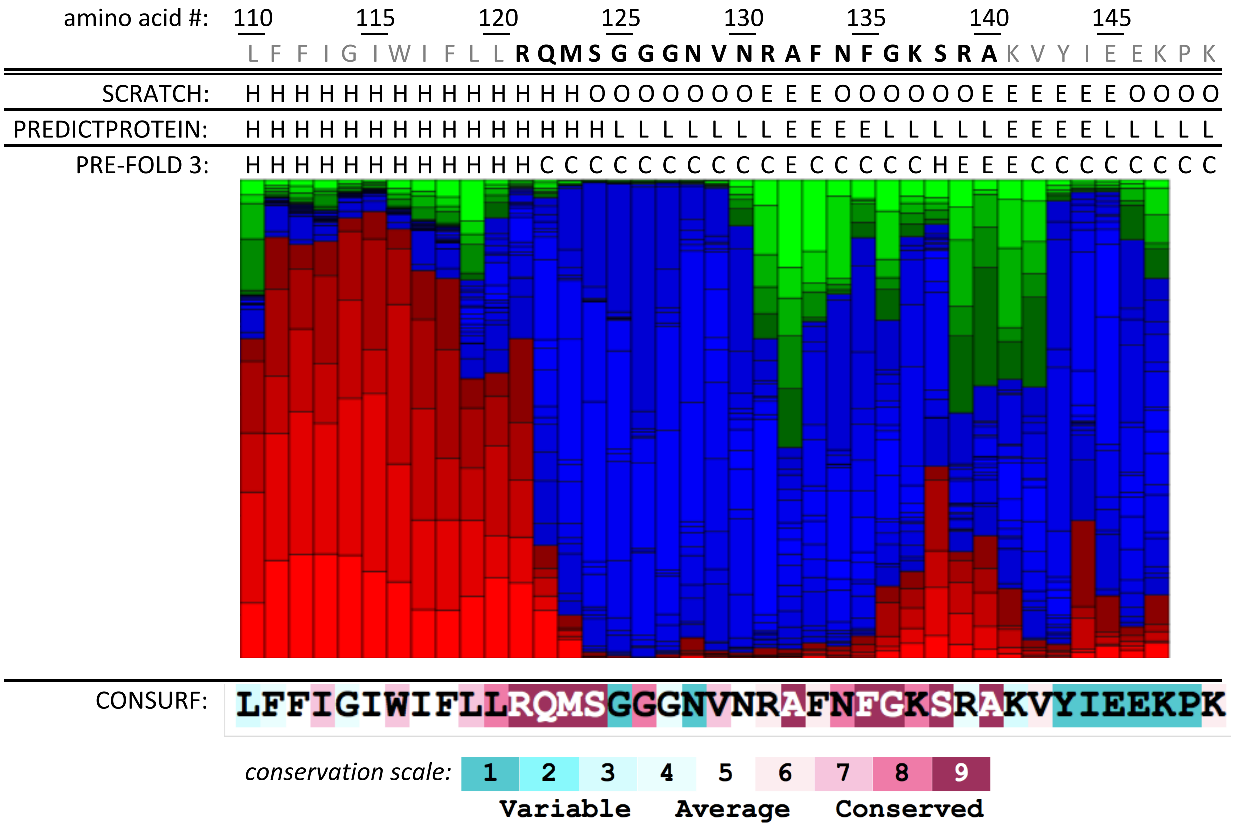

Figure S7. ~20 aa linker structure prediction. Structure of the ~20 aa region (amino acids in black) between the TM2 and the AAA-domain of *Aquifex aeolicus* FtsH, as predicted by the structure predictors SCRATCH, PREDICTPROTEIN and PRE-FOLD 3 (H: helical; E: extended; L: loop; C: coil; O: other). Residue conservation scores are obtained from the ConSurf server (scale at the bottom).

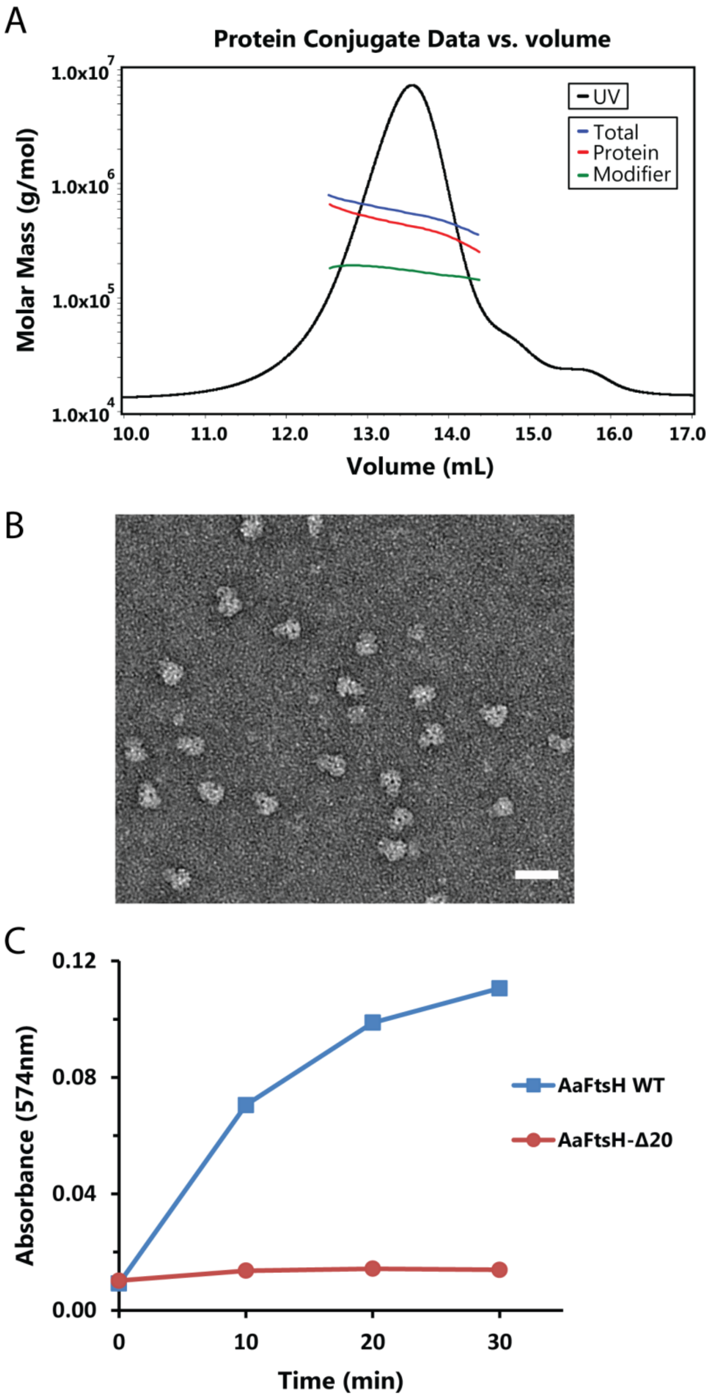

Figure S8. AaFtsH-Δ20 mutant characterization. *A* – SEC-MALS analysis of AaFtsH-Δ20. UV absorption chromatogram showing the distribution of the molar mass (g/mol) vs. retention volume (ml). The calculated molar masses of protein and detergent micelle are shown in supplementary Table S2. *B* – Negative stain EM of AaFtsH-Δ20 showing the hexameric assembly (side views). Scale bar is 200 Å. *C* – Protease activity assays. Resorufin release was measured over the course of 30 minutes for 50 μM of Resorufin labelled casein incubated with 1 μM AaFtsH-Δ20 mutant or AaFtsH wildtype.

Table S1. Data derived from SEC-MALS of AaFtsH

Peak	1*	2*
Total MW (kDa)	1096 ± 0.1 %	645 ± 0.2 %
Protein MW (kDa)	810 ± 0.4 %	427 ± 0.5 %
Micelle MW (kDa)	286 ± 0.6 %	218 ± 0.8 %

*Peak definition in Figure S1.

Table S2. Data derived from SEC-MALS of AaFtsH-Δ20 mutant

Total MW (kDa)	602 ± 0.2 %
Protein MW (kDa)	416 ± 0.4 %
Micelle MW (kDa)	186 ± 0.4 %

Table S3. Primers used in this study

Primer	Sequence 5'-3'
AaFtsH _Primer 1	GATGAACATGCCCGGTTACTGGAACGTTGTGAGGGTAAACA
AaFtsH _Primer 2	GGTTTTTCCTCTATGTAAACCTTGAGGAGGAATATCCATATACCGATAAAG
AaFtsH _Primer 3	TGTTTACCCTCACAACGTTCCAGTAACCGGGCATGTTCATC
AaFtsH _Primer 4	CGGTATATGGATATTCCTCCTCAAGGTTTACATAGAGGAAAAACCG

Table S4. FtsH image acquisition and image processing

	Hexamers		Dodecamers			
Nominal magnification	130000 x		215000 x			
Pixel size (A)	1.05		0.64			
Total dose (e^{-}/A^{2})	80		53			
Exposure time (s)	12		7			
Frames per movie	60		35			
Images acquired	1371		3993			
Initially detected particles	35048		101726			
Particles entering 3D classification	7635		41818			
	**C6 symmetry**	**C1 symmetry**	**Intertwined**	**Lamellar**	**Touching**	**V-shaped**
Particles in final refinement	5649	2129	4351	2651	1722	3246
Resolution in Å ($FSC_{0.143}$)	6.6	15.9	17	19.5	20.5	12.3

Table S5. Dimensions of FtsH subunit

Negative stain 2D class averages		Cryo-EM 3D maps
**Dimensions of hexamers (Å) (SD, N=10)**		**Hexamer in C6 symmetry (Å)**
Protein length	167 ± 5	134
Cytosolic domain height	83 ± 7	78
Cytosolic domain width	131 ± 7	140
Periplasmic domain height	31 ± 3	28
Periplasmic domain width	63 ± 6	80
Micelles thickness	40 ± 4	40
Micelles width	100 ± 18	90
**Dimensions of dodecamers (Å) (SD, N=10)**		**Dodecamer**
Protein length	243 ± 8	290
Micelles thickness	43 ± 2	40
Micelles width	126 ± 7	138
